# Research Trends in Antimicrobial Oral Hygiene Products, the Oral Microbiome, and Dental Biofilm: A Bibliometric Analysis (2006–2025)

**DOI:** 10.3390/antibiotics15090839

**Published:** 2026-08-29

**Authors:** Adela Baca-García, Pilar Baca, Adela Abellán, María Teresa Arias-Moliz, Pilar Valderrama

**Affiliations:** 1 Department of Stomatology, School of Dentistry, University of Granada, 18071 Granada, Spain; abgbaca@ugr.es; 2 Instituto de Investigación Biosanitaria ibs., 18012 Granada, Spain; mtarias@ugr.es; 3 Microbiology and Parasitology Service, University Hospital of Getafe, 28905 Getafe, Spain; 4 Department of Microbiology, University of Granada, 18071 Granada, Spain; 5 Department of Economics, International University of La Rioja, 26006 Logroño, Spain; mariadelpilar.valderramabaca@unir.net; 6 Evaluation of Science and Scholarly Communication, Research Group, Faculty of Communication and Documentation, University of Granada, 18071 Granada, Spain

**Keywords:** microbiota, dental plaque, mouthwashes, chlorhexidine, dysbiosis, bibliometrics

## Abstract

*Objective*: This study aims to provide a global landscape of research into oral hygiene products with antimicrobial or microbiome-modulating activity through a comprehensive bibliometric analysis to identify trends and hotspots that may influence future research frontiers. *Methods*: A structured bibliographic search was conducted within the Web of Science Core Collection database from 2006 to 2025. Manual screening was performed to exclude duplicate records, studies that did not align with the core topic, and those failing to meet the predefined inclusion criteria. Bibliometric and visual analyses were performed using VOSviewer, CiteSpace, and the R package ‘bibliometrix’ to evaluate production metrics, citation networks, and multi-level collaboration patterns. *Results*: The analysis included 1007 publications. Sreenivasan PK was the most productive author, and Lundberg JO was the most cited. The United States, followed by India, Brazil, and China, led global research volume, while the United Kingdom and the Netherlands led in total citations. The *International Journal of Dental Hygiene* was the most productive journal (*n* = 48), and the *Journal of Dentistry* was the most cited (*n* = 1356). Burgeoning research hotspots include the impact of mouthwashes on the oral microbiome and systemic disorders, the controlled clinical use of chlorhexidine, and alternative formulations incorporating probiotics, herbal extracts, or hyaluronic acid. *Conclusions*: This study underscores a global shift in dental research priorities from traditional bacterial elimination toward preserving oral microbiota eubiosis. While chlorhexidine remains a subject of research due to its widespread use for therapeutic benefits, bibliometric research highlights its potential systemic consequences as a hotspot. Therefore, future research should focus on innovative antimicrobial formulations for mouthwashes and toothpastes that maintain oral health without causing dysbiosis.

## 1. Introduction

The oral microbiome comprises the entire ecosystem of the oral cavity, including microbes and their metabolites, all the genetic material, and the surrounding microenvironment, while the oral microbiota represents the microbial community [1,2]. It is the second most diverse and abundant microbial community in the human body after the gut. It is estimated that over 700 bacterial species are present in the oral cavity, with a composition dominated primarily by the *Firmicutes* and *Bacteroidota* phyla, which together can represent up to 70–80% of the total microbial community [2].

Oral biofilm or dental plaque originates from a salivary pellicle colonised by commensal microbes, developing into a complex, multi-species structure protected by an extracellular matrix [2]. Under normal conditions, it acts as a natural barrier that prevents pathogen growth and maintains dynamic homeostasis [2,3]. However, various determinants can disrupt this balance, shifting the oral microbiome from a symbiotic state to dysbiosis and pathogenicity.

Caries and periodontal disease are highlighted in investigations as they are the world’s most common chronic oral infections and the primary cause of tooth loss. Specifically, dental caries stems from ecological shifts in dental biofilms, where the high-frequency intake of fermentable carbohydrates, particularly sucrose, drives the dominance of acid-producing species like *Streptococcus mutans*, *Lactobacillus*, *Actinomyces*, *Bifidobacterium*, and *Veillonella*, outcompeting acid-sensitive commensals [4]. Regarding periodontal and peri-implant diseases, bacteria are essential, as evidenced by the reversibility of gingivitis through plaque removal. However, current evidence strongly suggests that periodontitis is not merely an infection caused by specific pathogens, but an inflammatory disease driven by a dysregulated host immune response to microbial dysbiosis [5].

Beyond local pathologies, oral dysbiosis—particularly via periodontitis—establishes a state of chronic, localised inflammation that may influence systemic health through several mechanisms, including the haematogenous dissemination of oral pathogens and virulence factors like lipopolysaccharides, chronic systemic inflammation, molecular mimicry in autoimmune disorders, and microbial metabolic by-products [6]. Consequently, evidence supports strong links between oral dysbiosis and several chronic non-communicable conditions, including atherosclerotic cardiovascular disease [7], the bidirectional pathological link of periodontitis with both Type 2 Diabetes Mellitus [8] and inflammatory bowel disease [9], neurodegenerative conditions such as Alzheimer’s disease [10], rheumatoid arthritis [11], colorectal cancer [12], respiratory diseases [13], and potential associations with pregnancy-related complications [14].

In dental plaque control, the use of mechanical oral hygiene devices is essential for their removal. Additionally, chemical agents with antimicrobial activity are widely recognised for maintaining hygiene by reducing microbial load [15]; these are used preferably alongside mechanical methods, though also independently.

Chlorhexidine (CHX) is the most frequent plaque-control antimicrobial due to its broad-spectrum, long-lasting activity [16]. Short-term use prevents plaque and reduces gingivitis [17], making CHX mouthwash common in ambulatory periodontal and peri-implantitis treatments to limit biofilm, inflammation, and bleeding [18,19]. However, its efficacy triggers side effects like brownish discolouration, mucosal lesions, bad taste [20], or hypersensitivity [21]. Next-generation sequencing shows CHX mouthwash reduces bacterial diversity and decreases species essential for oral and systemic health, particularly nitrate-reducing bacteria [16].

Like CHX, cetylpyridinium chloride and essential oils possess broad antimicrobial potential [22]. A recent systematic review [23] showed CHX caused the greatest dysbiosis, reducing diversity by 40–60%; cetylpyridinium chloride preserved 70–80% of commensals, while herbal rinses reduced pathogens by 25–40% without disrupting balance. Conventional antimicrobials incur this bystander damage, which may have systemic consequences [24,25]. Decreasing nitrate-reducing bacteria impairs nitric oxide production, potentially elevating blood pressure [26,27,28]. Therefore, alternative oral antiseptics promoting a healthy ecosystem instead of dysbiosis are required [29].

Bibliometric analysis identifies research characteristics and trends within specific fields. Several studies have evaluated the oral microbiome bibliometrically, revealing a rapidly expanding landscape that emphasises dysbiosis [30] and its interplay with systemic diseases, immunity, and cancer [31]. Bibliometric research on the oral microbiome–cancer axis highlights a hotspot focused on dysbiosis and cancer development [32,33], alongside growing interest in mouthwash utilisation for mucositis and cancer therapy [34]. Regarding periodontitis, recent thematic evolution analyses indicate a paradigm shift from a single-pathogen model, exemplified by *Porphyromonas gingivalis*, to an ecological framework focused on microbial dysbiosis, whilst also highlighting microbiome-based therapeutic strategies [35]. Furthermore, trends in clinical trials focused on oral biofilm have been examined [36]. Several agents with antimicrobial activity have also been analysed in relation to dental biofilm. A bibliometric and critical review of randomised clinical trials concluded that probiotics and dairy products offer promising alternatives for human oral parameters [37]. Similarly, data on Chinese herbal medicine mouthwash reveal growing interest and more comparative trials against chemical options [38]. Most recently, a bibliometric analysis has also evaluated the global role of CHX in dentistry, restricting its scope to the Dentistry, Oral Surgery, and Medicine category [39].

However, to the best of our knowledge, no previous study has conducted a bibliometric analysis of antimicrobial agents in oral care products without limiting the study design. Therefore, this study aims to provide a global landscape of research into oral hygiene products with antimicrobial or microbiome-modulating activity, through a comprehensive bibliometric analysis to identify trends and hotspots that may influence future research frontiers.

## 2. Results

### 2.1. Publication Trends and Most Cited Documents

**Figure 1:**
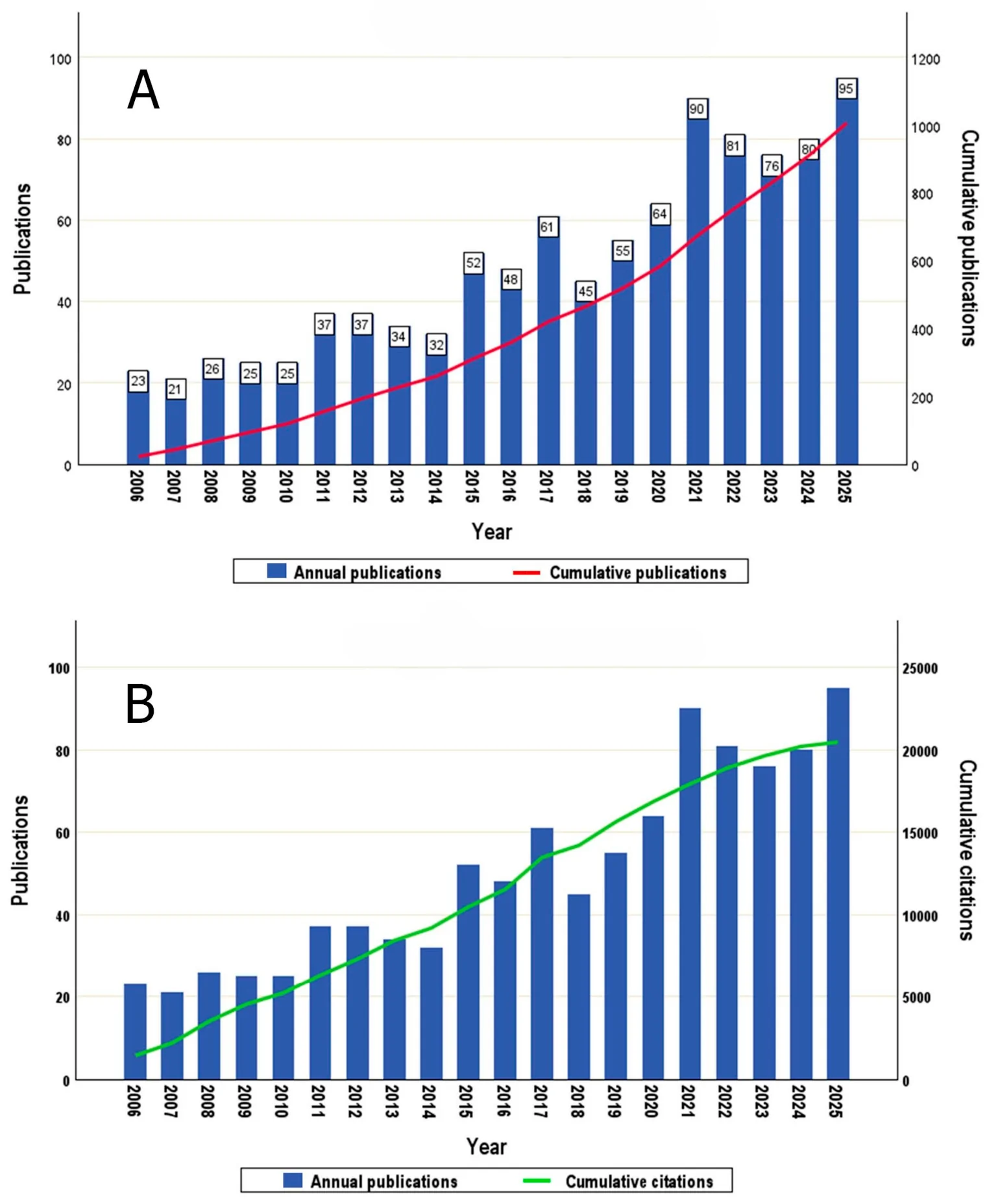
Publication and citation trajectories (2006–2025). (**A**) Annual publication volume and cumulative publications. (**B**) Cumulative citations growth over time.

Across the analysed timespan, the field achieved an Annual Growth Rate of 7.75%. As illustrated in Figure 1A,B, annual output rose from 23 publications in 2006 to 95 in 2025. Furthermore, cumulative publications and citations are shown.

The top ten most cited documents are listed in Table 1, led by Govoni et al. [40] with 511 citations (28.39 citations/year) for their study on antibacterial mouthwash and plasma nitrite. Prominent thematic fields among these highly cited papers focus on mouthwash-related systemic disorders and the clinical effectiveness of CHX.

### 2.2. Author, Country, Institutional, and Journal Analysis

**Figure 2:**
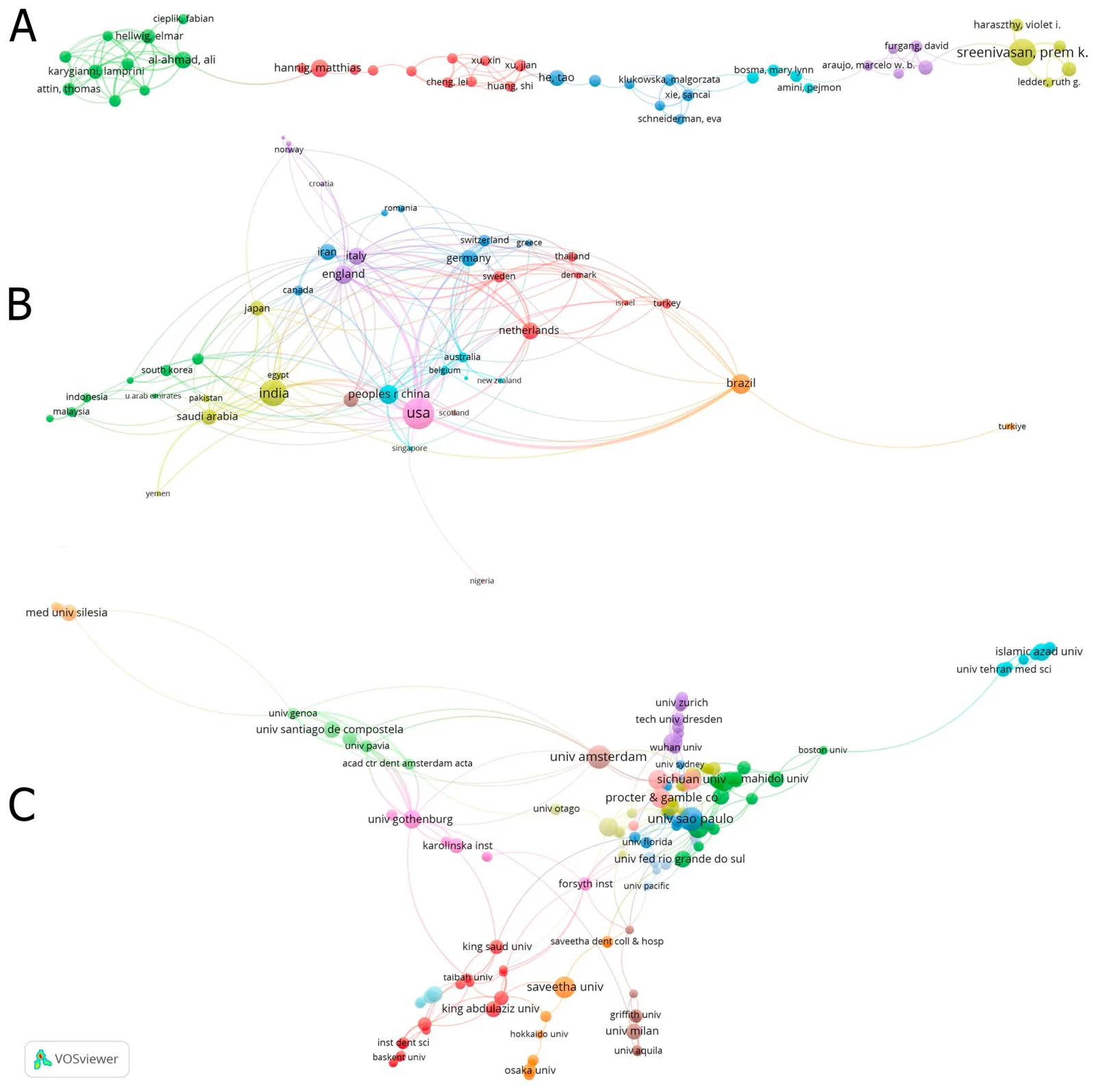
Co-authorship network maps for the 1007 retrieved documents. Collaboration networks across (**A**) authors, (**B**) countries, and (**C**) institutions based on a minimum threshold of ≥3 documents. Node sizes correspond to publication volume, line thickness reflects total link strength, and colours represent distinct collaborative clusters.

Table 2 details the top ten most productive authors, led by Sreenivasan PK (Colgate-Palmolive Technology Center, USA) with 28 articles (459 citations). This volume is driven by three co-authoring clusters from Vrije Universiteit Amsterdam, Universidad Complutense de Madrid, and the University of Groningen. Notably, the most cited researchers are not the most productive. At the Karolinska Institutet (Sweden), Lundberg JO (5 documents) co-authored the 1st, 3rd, and 9th most cited papers in Table 1; Weitzberg E (4 documents) contributed to the 1st and 3rd; and Jansson EA (2 documents) to the 1st and 9th. Additionally, Govoni M first-authored the most cited paper (1 document), while the authors of the second most cited article also published just one document within the 1007-paper dataset. The author collaboration network, depicted in Figure 2A, revealed six relatively disconnected clusters.

**Figure 3:**
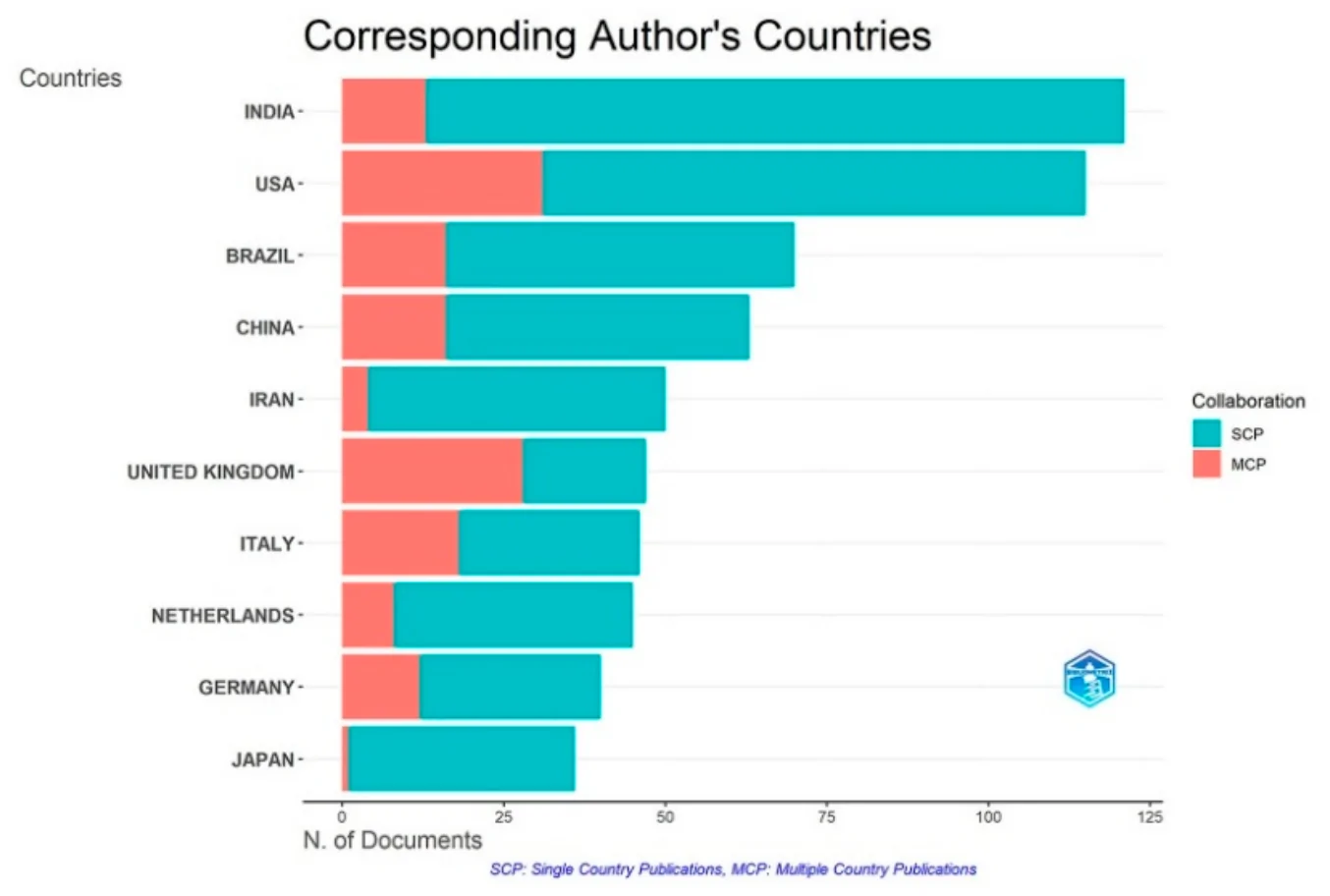
Global publication volume according to the corresponding author’s country, distinguishing between Single Country Publications (SCP) and Multiple Country Publications (MCP).

Table 3 lists the top ten most productive countries, led by the United States with the highest output (187 documents) and citations (4621). India (138), Brazil (78), and China (69) followed in productivity, while the United Kingdom (2662) and the Netherlands (1651) ranked next in citation counts. Figure 2B shows international collaboration remains partially regional, featuring a Euro-Asian cluster led by the Netherlands and an Asian cluster led by India. Lastly, Figure 3 reveals that single-country publications predominated globally, with the United Kingdom as the sole exception.

Among the 1307 institutions identified, the University of Amsterdam (Netherlands) and the University of São Paulo (Brazil) led productivity with 22 documents each, followed by Vrije Universiteit Amsterdam (*n* = 19). Both Dutch universities maintained the highest citation counts (Table 4). Furthermore, the institutional collaboration network (Figure 2C) demonstrates sparse links and limited cooperation.

**Figure 4:**
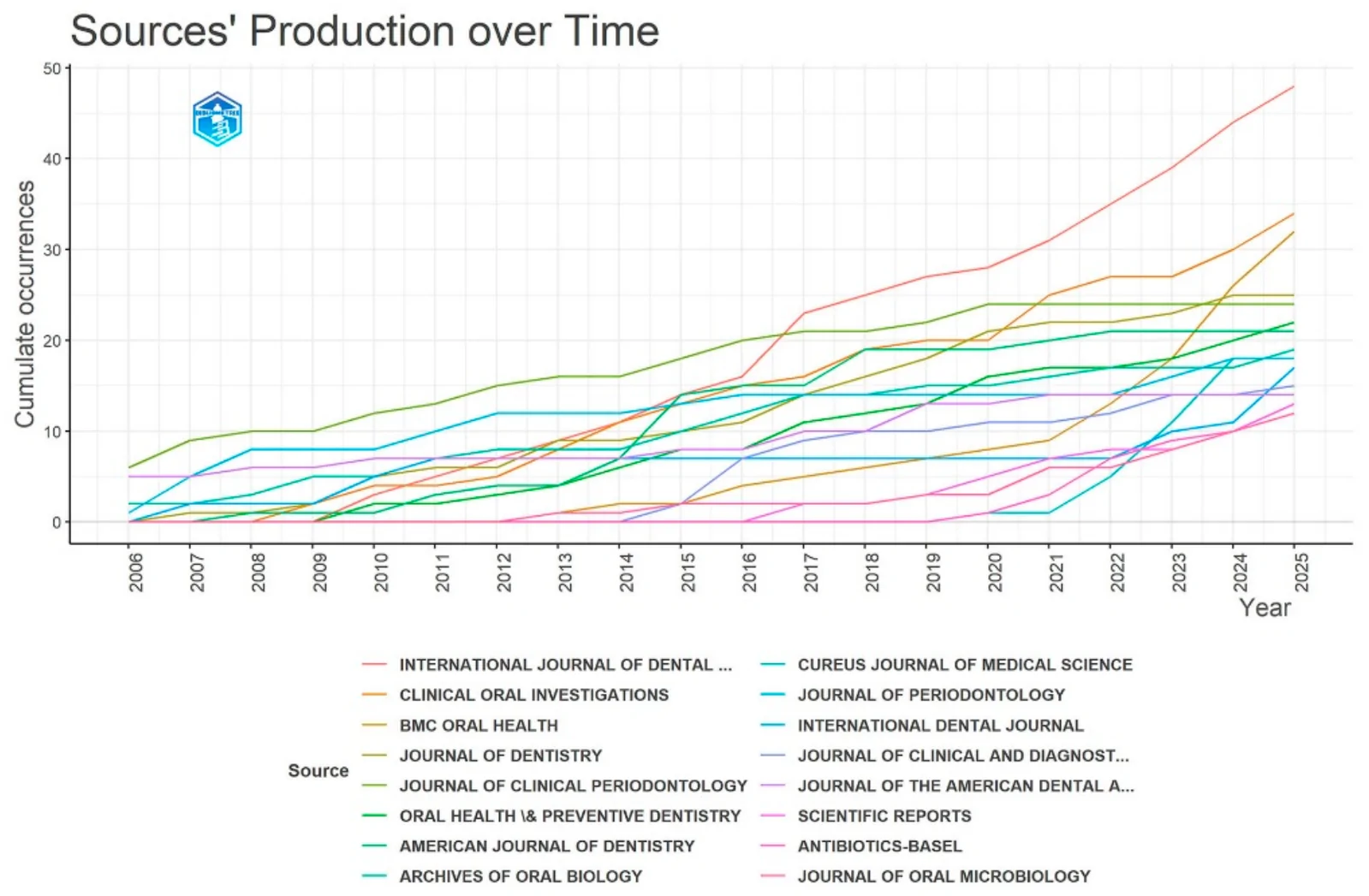
Annual cumulative publication output over time for the 16 core zone journals identified by Bradford’s Law.

The dataset spans 339 journals. According to Bradford’s law, core zone 1 comprises 16 journals (Table 5), eleven of which fall within the Dentistry, Oral Surgery, and Medicine category. Regarding Journal Citation Reports (2025) impact factor, ten are Q1, one is Q2, one is Q3, three are Q4, and one is unlisted. Notably, the *International Journal of Dental Hygiene* yielded the highest output (*n* = 48), whilst the *Journal of Dentistry* accumulated the most citations (*n* = 1356). Figure 4 illustrates the chronological growth of these 16 core journals.

### 2.3. Keywords, Trend Topics, and Thematic Evolution

**Figure 5:**
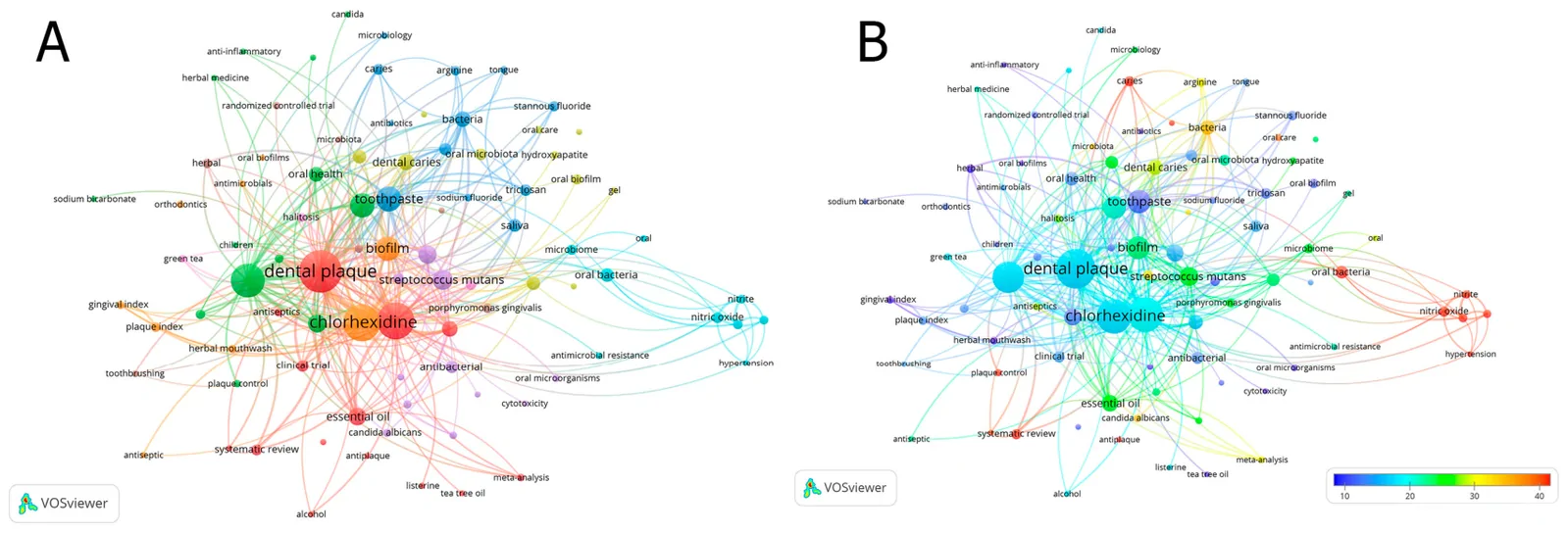
Keyword co-occurrence and trend topic analyses. (**A**) Network map of the 89 keywords meeting the ≥6 occurrences threshold, distributed across 7 keyword clusters. (**B**) Overlay visualisation where red/orange colours indicate high average citations (~50) and blue/violet represent the lowest. Node sizes reflect publication volume and line thickness indicates total link strength.

Table 6 lists the top 20 most frequent author keywords (minimum threshold: six occurrences). The keyword network (Figure 5A) is dominated by a central hub occupied by ‘dental plaque’ (293 occurrences), ‘chlorhexidine’ (*n* = 227), and ‘mouthwash’ (*n* = 224), all exhibiting extensive links across clusters. Furthermore, node colours reflect citation levels (Figure 5B); concepts such as ‘nitrite’, ‘nitric oxide’, and ‘hypertension’ are represented by smaller yet highly cited nodes, similar to systematic reviews.

**Figure 6:**
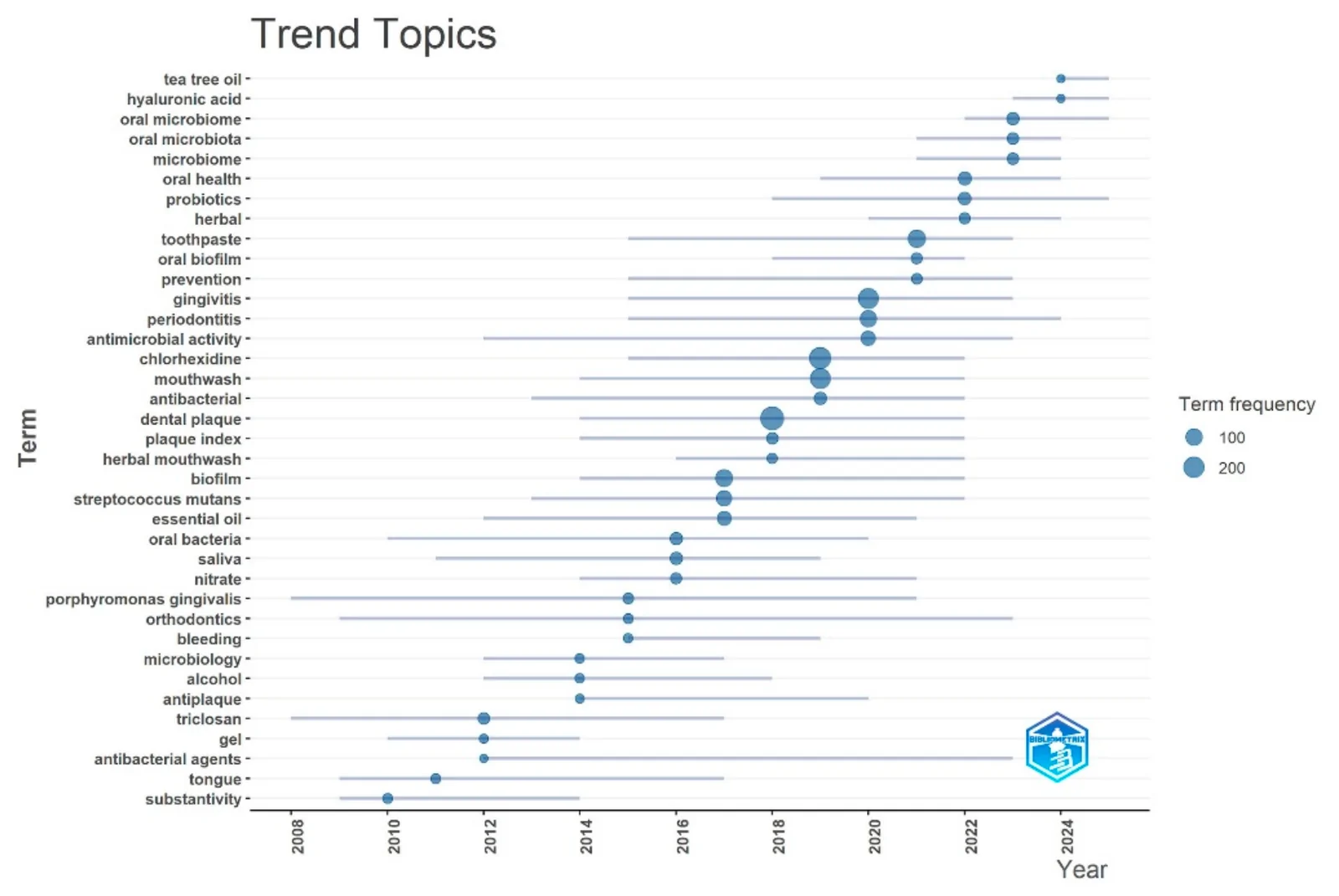
Trend topic plot mapping the chronological evolution and prevalence of top research terms over time, with node sizes proportional to keyword frequency across publication years.

A trend topic plot (Figure 6) illustrates the chronological evolution of author keywords, selecting the top three most frequent keywords appearing within each specific year.

**Figure 7:**
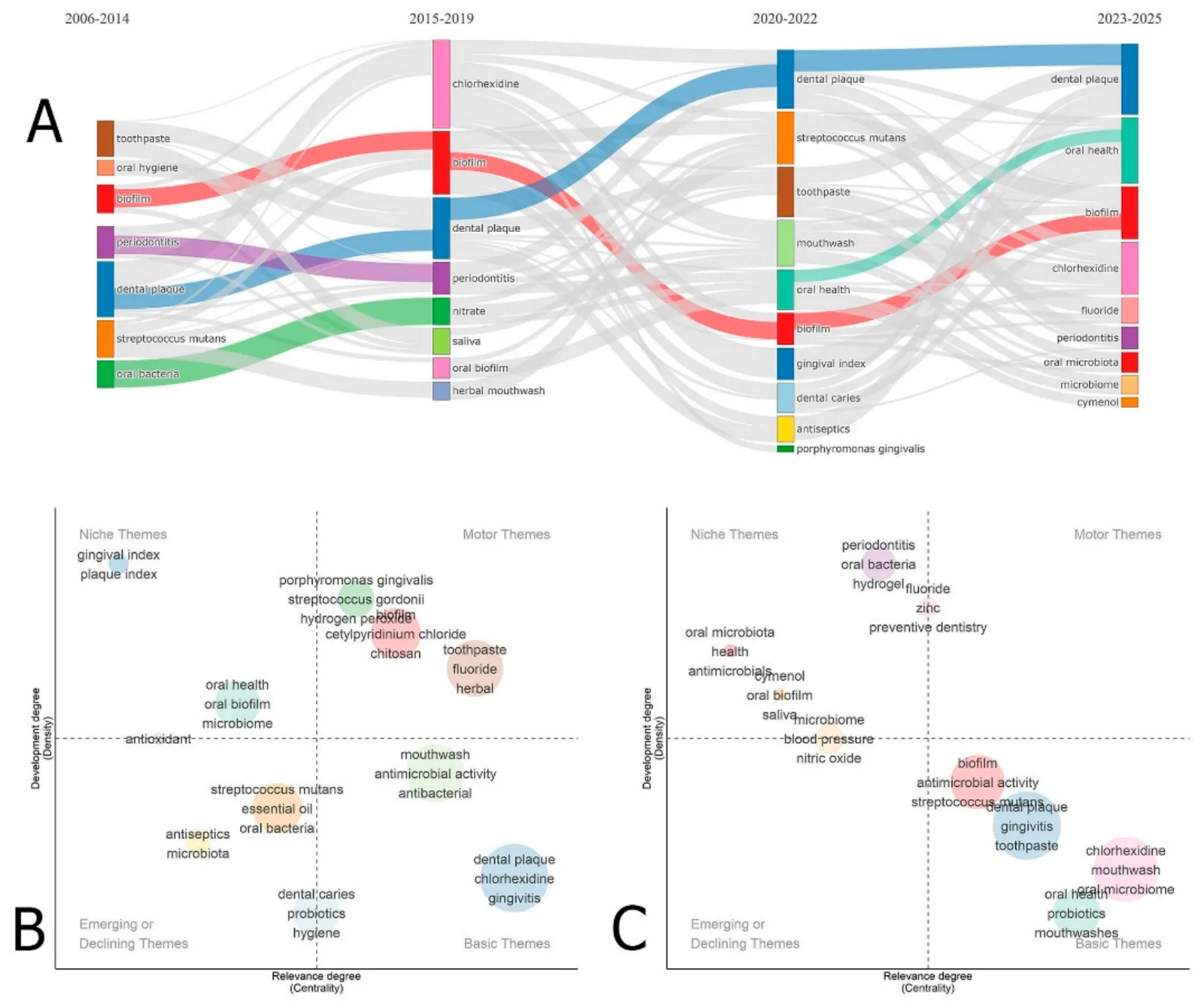
Thematic evolution and strategic analyses of the retrieved documents. (**A**) Alluvial diagram illustrating the chronological transformation, split, and convergence of research themes across four consecutive sub-periods (2006–2014, 2015–2019, 2020–2022, 2023–2025), where vertical blocks represent clusters and connecting flows indicate structural continuity. (**B**) Strategic diagram (2020–2022) categorising research themes into motor, niche, emerging/declining, and basic quadrants based on keyword centrality (x-axis) and density (y-axis). (**C**) Strategic diagram (2023–2025) mapping thematic distribution in the most recent period using the same quadrant classification.

The thematic evolution across four document-balanced periods (Figure 7A) reveals the persistence of core themes; notably, ‘dental plaque’ and ‘biofilm’ remain present throughout. Other keywords, such as ‘periodontitis’, feature consistently across multiple time slices, whilst high-volume dominant themes like ‘chlorhexidine’ are represented by thick blocks. Furthermore, the analysis highlights the recent rise of ‘microbiome’ and ‘oral microbiota’, alongside emerging compounds such as ‘cymenol’.

Figure 7B categorises the thematic map during the 2020–2022 sub-period into four strategic quadrants. Within Motor themes, active formulations multiply, featuring compounds like ‘hydrogen peroxide’, ‘cetylpyridinium chloride’, ‘fluoride’, and ‘chitosan’. Basic themes include ‘dental plaque’, ‘chlorhexidine’, ‘gingivitis’, and ‘mouthwash’, and Niche themes include keywords such as ‘microbiome’ and ‘oral health’. Concurrently, the emerging or declining themes include research focused on ‘*Streptococcus mutans*’, ‘caries’, ‘microbiota ’, and ‘essential oil’. Finally, the 2023–2025 sub-period (Figure 7C) shows that ‘oral microbiome’, ‘oral health’, and ‘probiotics’ have been incorporated into Basic themes alongside habitual terms: ‘chlorhexidine’, ‘dental plaque’, ‘mouthwash’, and ‘gingivitis’.

### 2.4. Reference Co-Citation Analysis

**Figure 8:**
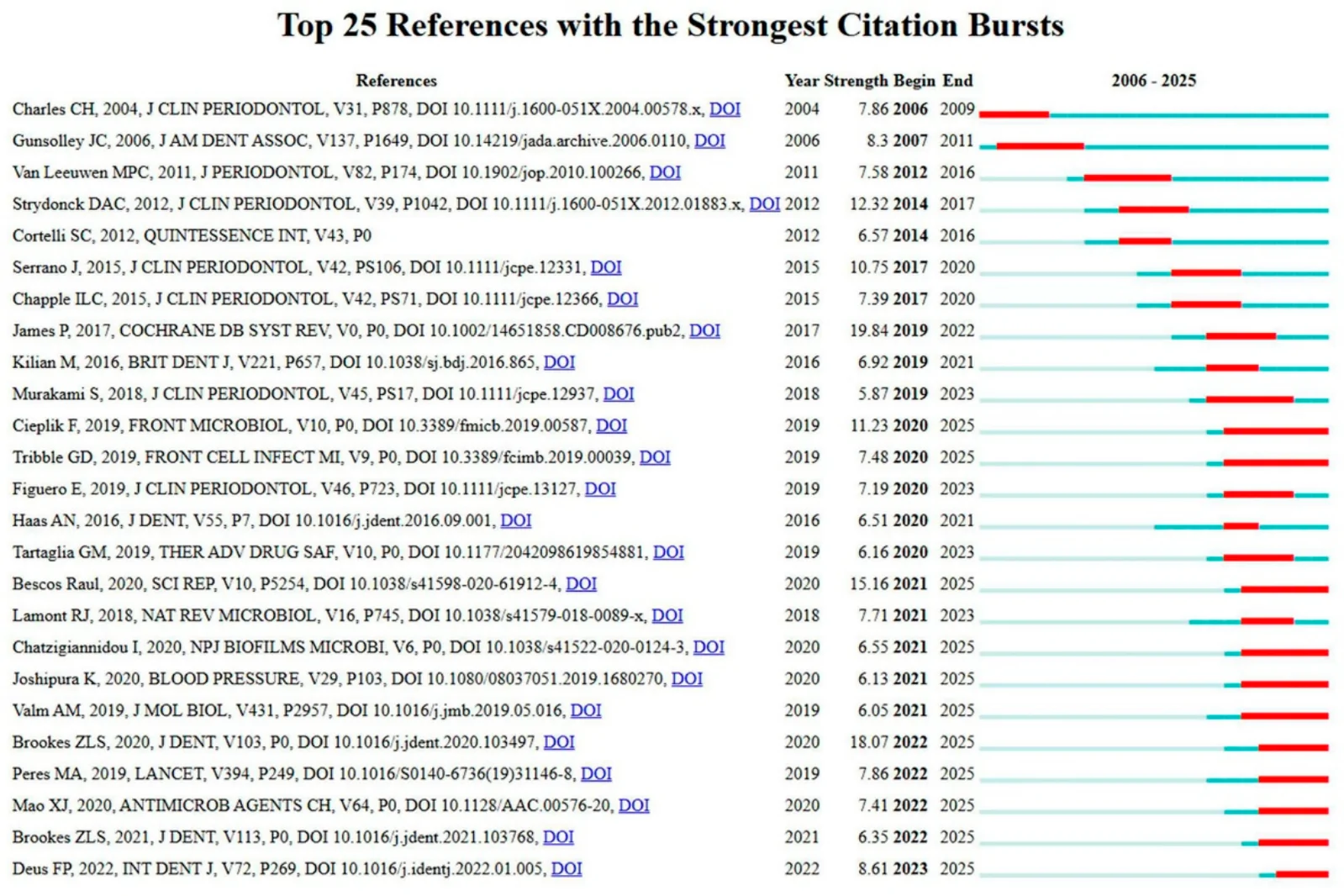
Top 25 references with the strongest citation bursts (2006–2025). Timeline tracks annual publication lifespan (thin blue lines), pre-burst periods (thick blue lines), and exact burst durations (red segments); bursts extending to 2025 identify the current research front.

The citation burst analysis presented in Figure 8 identified the references that generated a substantial impact on the field during the 2006–2025 period. Notably, three key references emerged: James et al. (2017) [18] (Strength: 19.84, begin 2019–end 2022), Brookes et al. (2020) [41] (Strength: 18.07, begin 2022–end 2025), and Bescos et al. (2020) [42] (Strength: 15.16, begin 2021–end 2025).

**Figure 9:**
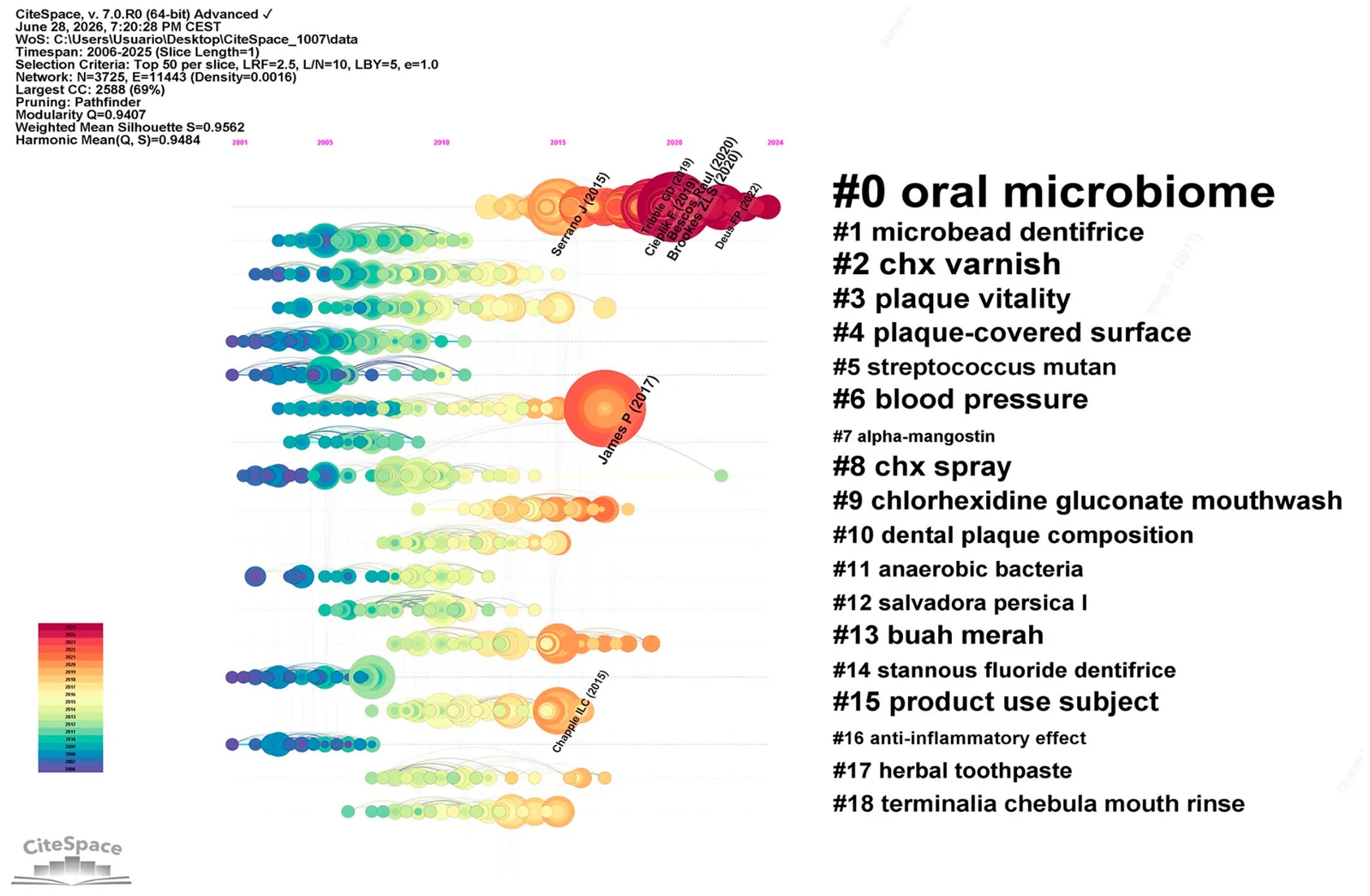
Reference co-citation timeline view configured with one-year time slicing (top 50 selection criterion per slice) and optimised using the Pathfinder pruning algorithm. Horizontal lanes represent thematic clusters labelled via Log-Likelihood Ratio (LLR) analysis. Node diameters reflect cumulative citation volume, and colouring denotes chronological periods from cool to warm tones.

The reference co-citation network achieved a well-defined clustering, as mathematically validated by the robust Modularity (Q = 0.9407 > 0.3) and Weighted Mean Silhouette (S = 0.9562 > 0.7) scores. Although the complete network identified 41 clusters in total (detailed in Supplementary Table S1), the timeline view focuses on the 19 major clusters (numbered #0 to #18) that meet CiteSpace’s automated visualisation thresholds (Figure 9), ranging in size from 82 to 11 items. This structure was optimised using the Pathfinder pruning algorithm to remove minor peripheral components. The largest and one of the most recent groups is Cluster #0 (size = 82, mean year = 2018), which focuses on the ‘oral microbiome’, ‘oral health’, ‘oral pathogen’, and ‘systemic health’.

## 3. Discussion

This bibliometric analysis of antimicrobial oral hygiene products on the oral microbiome and dental plaque highlighted the dynamic growth, hotspots, and future trends in this field across the 2006–2025 period; these findings map the conceptual shifts in biofilm management and their systemic implications. The dataset encompasses a total of 1007 documents published over a 20-year period (2006–2025). This sample size meets the methodological thresholds required to ensure statistical stability in science mapping and clustering techniques [43]. The field achieved an Annual Growth Rate of 7.75% during this period. Although Figure 1A,B displays a steady rising trend in cumulative publications and citations, this pattern primarily reflects the continuous structural accumulation of data over time rather than a formal acceleration of research activity. Nevertheless, this annual growth rate remains highly notable, as it consistently outpaces the typical 4% to 5.5% expansion traditionally observed across global scientific literature [44].

The author collaboration network (Figure 2A) indicates sparse cooperation between distinct research groups, although several prominent clusters emerge (Table 4).

Individually, Sreenivasan PK (Colgate, USA) led in publication volume (*n* = 28), but he was not the most cited. Conversely, European networks dominate scientific impact through highly prolific research groups. Van Der Weijden GA and Slot DE of Vrije Universiteit Amsterdam co-authored 14 documents, including a landmark systematic review on CHX efficacy [20] that establishes this Dutch team as a benchmark for oral hygiene trials. Next, Herrera D, alongside Sanz M and Serrano J (Complutense University of Madrid, Spain), excels in antimicrobial clinical trials and coordinated the European Federation of Periodontology (EFP) treatment guidelines [45]. Finally, Busscher HJ and Van Der Mei HC of the University of Groningen (the Netherlands) co-authored 12 papers, specialising in antimicrobial efficiency within biofilm models. By citation impact, four co-authors from the Karolinska Institutet (Sweden) stand out; despite low publication volumes, their scientific repercussion is substantial. Lundberg JO (1082 citations, 5 papers) co-authored three of the top ten most-cited documents (ranks 1, 3, and 9) (Table 1), whilst Weitzberg E and Jansson EA contributed to two each. Govoni M was first author on the top-ranked paper (511 citations). These studies indicated how antimicrobial mouthwashes disrupt the nitrate–nitrite–nitric oxide pathway and blood pressure regulation. This systemic implication drives the high citations across a few publications, positioning them as highly influential rather than merely prolific authors. This cohort exemplifies the dataset’s dichotomy: highly productive authors versus those with limited output but a high citation impact.

The United States was the most productive country in terms of publication output (Table 3), a common trend across many research fields, and it has recently demonstrated the highest publication volume in oral microbiome studies [30]. Furthermore, Figure 2B demonstrates extensive US partnerships with prominent countries in the network, an output which may be influenced by industrial research trends; both Colgate-Palmolive and Procter & Gamble rank among the top ten most productive institutions, and the most prolific author, Sreenivasan PK, is affiliated with Colgate-Palmolive. Conversely, India ranks as the second most productive nation but exhibits the lowest citations per document (10.08), highlighting a stark recurrent divergence between research volume and scientific impact (Table 3). This pattern is replicated in the institutional network, where the University of São Paulo ranks as the second most productive institution despite a modest citations per document (12.68). In contrast, the United Kingdom ranks fifth in production but leads in citations per document (40.95); British authors, including Kapil V, Brookes ZL, and Bescos R, have authored some of the most cited documents and highest-strength citation bursts regarding the oral microbiome and CHX (Figure 8).

In terms of institutional output, two Dutch universities stand out in the first and third positions, respectively: the University of Amsterdam and Vrije Universiteit Amsterdam. This strong performance aligns with the fact that the Netherlands ranks as the second leading nation in citations per document (29.48). Although bibliometric metrics process them as distinct nodes, this trend may largely reflect the scientific production of the Academic Centre for Dentistry Amsterdam (ACTA), to which both universities are affiliated, thereby positioning the Netherlands as a highly influential contributor to oral microbiome research.

Although most core zone journals are in Dentistry (11 out of 16), the inclusion of other categories such as General and Internal Medicine, Infectious Diseases, and Microbiology highlights a notable breadth of topics, which may indicate an interdisciplinary interest surrounding the CHX-oral microbiome–systemic health axis (Table 5). The temporal analysis (Figure 4) tracks a steady increase in output across these core journals from 2006 to 2025; whilst most grow continuously, some exhibit periods of stagnation, which might suggest potential shifts in editorial focus. Since 2017, the *International Journal of Dental Hygiene* stands out with the most pronounced rise.

To reliably capture research frontiers and emerging hotspots while mitigating software-specific biases, a multi-indicator framework using three complementary platforms was employed [46]. This methodological approach has been successfully utilised in recent bibliometric dental research [47,48]. Specifically, the strategy integrated overall citation metrics with keyword co-occurrence via VOSviewer to map the conceptual structure, trend topics, and thematic evolution through the R package ‘bibliometrix’ (Biblioshiny), and timeline references alongside citation burst detection using CiteSpace to trace the temporal trajectory of scientific knowledge.

The most cited research in the dataset establishes a clear clinical profile for CHX (Table 1). The first [40] and third [49] most cited papers (511 and 321 citations) demonstrate through clinical designs that CHX disrupts the symbiotic nitrate–nitrite–nitric oxide pathway by suppressing oral microflora. This reduction in nitric oxide bioavailability may influence blood-pressure regulation, suggesting that oral bacteria may play a vital role in cardiovascular health. The second most cited paper (399 citations; 44.33 citations/year) [18] provides meta-analytic evidence from 51 studies confirming that while CHX effectively reduces dental plaque and mild gingivitis, prolonged use over four weeks triggers adverse effects like extrinsic tooth staining and taste disturbances. This study had a disruptive and transient impact on this field of study from 2019 to 2022, achieving the highest strength (19.84) among the top references with the strongest citation bursts (Figure 8). By citations per year, the top paper (198 citations; 2024) [50] is a review on oral microbiome impact on oral and systemic health, exploring links to systemic diseases and its modulation via various therapies. The second (273 citations; 45.50 citations/year) [40] reviews current CHX uses for plaque and gingivitis control. These articles highlight the paradox of broad-spectrum mouthrinses: despite their proven efficacy against dental plaque, the literature raises concerns regarding potential systemic implications. Visually, a cluster with higher citations can be seen in Figure 5B in red, which includes ‘nitrite’, ‘nitric oxide’ and ‘hypertension’, with potential systemic implications connected with ‘oral bacteria’.

Keyword analysis constitutes a robust method for tracking clinical trends and systematically extracting conceptual shifts over time [51]. In this study, the most frequent keywords (Table 6) are headed by generalist terms such as ‘dental plaque’, ‘mouthwash’, and ‘gingivitis’, which capture the thematic core of research in this field, as evidenced in Figure 5A. However, when examining specific active ingredients, ‘chlorhexidine’ (*n* = 227) is firmly positioned at the forefront, being the primary adjunctive antiseptic recommended by key periodontal consensus workshops [18,44]. This is followed by ‘essential oils’ and ‘cetylpyridinium chloride’ within the top twenty keywords, consolidating themselves as the most documented clinical alternatives [52], particularly when the prolonged use of CHX is restricted by its well-known adverse effects. Right below these conventional agents, the terms ‘probiotic’ and ‘oral microbiome’ occupy the 17th and 18th positions in the ranking, quantitatively confirming a bibliometric conceptual shift oriented toward ecological modulation and the preservation of oral microflora symbiosis.

The evolution of keywords over time allows a broader analysis. Figure 6 illustrates terms appearing as top keywords in specific years, where node size reflects relative prominence. The four highest-frequency keywords—‘dental plaque’, ‘chlorhexidine’, ‘mouthwash’, and ‘gingivitis’—are confirmed as central hubs (node size ≥ 200). However, declining topics are visible; for instance, ‘substantivity’ emerged around 2010 but appears superseded by newer concepts. Concurrently, emerging topics have gained prominence in recent years, such as ‘oral microbiome’ and ‘hyaluronic acid’, rising from 2022 and 2023 onwards. Furthermore, the data confirms that these are expanding research fields, as ‘probiotics’, ‘oral health’, ‘oral microbiome’, and ‘oral microbiota’ are represented by large nodes in the final years of the dataset (2022–2023). A topic-specific analysis reveals that ‘triclosan’ appeared between 2008 and 2017, peaking in 2012, but its relevance in contemporary research is scarce. Conversely, ‘essential oils’ and ‘herbal’ compounds remain influential, with ‘tea tree oil’ and ‘hyaluronic acid’ emerging as very recent topics. Notably, these two agents, alongside ‘probiotics’ and the ‘oral microbiome’, remained at the forefront until the end of the evaluated period.

The analysis of thematic evolution (Figure 7A–C) maintains the consistency of terms such as ‘dental plaque’ and ‘biofilm’ present throughout all periods, while displaying dominant positions for ‘chlorhexidine’ and ‘periodontitis’. Concurrently, it highlights the recent rise in ‘microbiome’ and ‘oral microbiota’ and identifies the recent emergence of a specific essential oil, ‘cymenol’—a phenolic compound derived from thymol that exhibits direct antimicrobial effects. The bibliometric analysis of the final sub-period (2023–2025) shown in Figure 7 C suggests a notable conceptual evolution, with the ‘oral microbiome’ and ‘probiotics’ establishing themselves as foundational pillars alongside ‘biofilms’ and traditional CHX-based mouthwashes.

The presence of ‘essential oils’ and ‘herbal’ suggests a growing interest in natural or alternative antimicrobial approaches in oral care. These options tend to offer a more biocompatible profile, mitigating the dysbiosis typically associated with chlorhexidine (CHX) [23]. Within this trend, the emergence of ‘tea tree oil’ stands out as a prominent topic. However, further research is required to standardise dosing and confirm long-term efficacy [53]. On the other hand, the essential oil cymenol with cetylpyridinium chloride has demonstrated no adverse effects on the composition of the subgingival microbiome [54].

Regarding probiotics, they have proven useful in reducing plaque and controlling periodontal disease [55], without the disruption caused by CHX [23]. However, the development of formulations, the improvement of delivery systems, the standardisation of protocols, and the assessment of the risks of genetic transfer in immunocompromised patients remain crucial areas for improvement [55].

Hyaluronic acid exerts a bacteriostatic effect and even possesses anti-biofilm properties in the periodontal environment [56]. It has shown utility in plaque and gingivitis control compared to CHX [57], as well as an effect on clinical periodontal parameters when used as an adjunct to sodium hypochlorite [58]. This biological versatility has directly driven its rise as a prominent emerging topic in the bibliometric mapping of recent years.

References with the strongest citation bursts represent key documents and pillars of current research, marking the structural shifts and thematic evolution within the field up to 2025 (Figure 8). Among those persisting into 2025, three achieve a burst strength greater than 10, illustrating the bibliometric transition from traditional CHX evaluation towards ecological paradigms. These include the narrative review by Brookes et al. (2020) [40] (strength 18.07) on contemporary CHX uses for plaque control; the clinical trial by Bescos et al. (2020) [42] (strength: 15.16), which demonstrated that CHX alters the salivary microbiome, shifting focus toward homeostasis; and, finally, the review by Cieplik (2019) [59] (strength: 11.23), which raises awareness within the dental community regarding CHX resistance and its cross-resistance to antibiotics. This underscores the fact that dental professionals must bear in mind that the oral microbiota represents a significant—yet still underexplored—reservoir of antimicrobial resistance genes [60]. Within this topic lies the article with the most recent citation burst published in 2022 by Poppolo Deus et al. [61] (strength: 8.61, begin 2023–end 2025) focuses anew on the pharmacology and potential adverse effects of CHX, reflecting sustained bibliometric interest in the limitations of standard treatments. In the timeline view of the reference co-citation analysis (Figure 9), cluster #0 ‘Oral microbiome’ stands out as the most recent and largest, and it includes all the previously highlighted references. This emerging interest in the oral microbiome is consistent across all analysed bibliometric indicators, reflecting a clear scientific drive in the literature to develop safer therapeutic alternatives [62] that preserve oral ecology without compromising effective biofilm control and dental health.

### Limitations

This bibliometric study is not without limitations, and the methodology employed must be considered when interpreting the results. First, the data were retrieved from the Web of Science Core Collection (WoSCC). Although this is a widely recognised database, publications indexed in alternative platforms or non-indexed literature may have been omitted, influencing the results and citation patterns. Furthermore, a specific search strategy was used. Although designed by experts and intended to balance sensitivity and specificity, the inclusion of the Boolean operator NOT may have missed several studies. In addition, studies published in languages other than English were excluded. All of these factors combined may have resulted in an incomplete dataset. Additionally, bibliometric indicators capture research patterns and citation structures, but they do not directly measure the intrinsic methodological quality of the articles or their clinical validity. Finally, the limitations of the software used (VOSviewer, CiteSpace, and R package ‘bibliometrix’) must be acknowledged, as their algorithms and applied filters can alter some thematic or keyword relationships.

## 4. Materials and Methods

### 4.1. Database and Search Strategy

For the present analysis, the search query was performed on 24 May 2026, without any restrictions, on the Web of Science Core Collection (WoSCC, Clarivate, Philadelphia, PA, USA). The search strategy was executed in the Topic field (TS=) employing a combination of five term sets. The final strategy was as follows: ((“mouthwash*” OR “mouth rinse*” OR “mouthrinse*” OR “rinse*” OR “dentifrice*” OR “toothpaste*” OR “gel*” OR “spray*” OR “dental floss” OR “dental silk” OR “oral irrigator” OR “water flosser” OR “dental water jet”) AND (“anti plaque” OR “antibacterial agent*” OR “antimicrobial” OR “chemical agent*” OR “antiseptic*” OR “chlorhexidine” OR “hexamidine” OR “alexidine” OR “essential oil*” OR “quaternary ammonium” OR “cetylpyridinium” OR “benzalkonium” OR “povidone iodine” OR “hydrogen peroxide” OR “listerine” OR “amine fluoride*” OR “stannous” OR “zinc” OR “detergent*” OR “herbal” OR “triclosan” OR “peptide*” OR “probiotic*” OR “prebiotic*” OR “propolis” OR “Hydrogen” OR “delmopinol” OR “curcum*” OR “arginine” OR “*hyaluronic*” OR “peroxide”) AND (“oral microbiota” OR “oral microbiome” OR “oral biofilm*” OR “oral bacteria*” OR “oral microorg*” OR “oral microflora” OR “oral flora” OR “oral dysbio*” OR “oral microenvironment*” OR “mouth microbiota*” OR “mouth microbiome” OR “mouth bacteria*” OR “salivary microbiota” OR “buccal microbiota” OR “dental microbiota” OR “dental microbiome” OR “dental plaque”)) NOT ((“gut” OR “intestinal” OR “nasopharyngeal” OR “nasal” OR “respiratory” OR “bronchial” OR “tracheal” OR “skin” OR “cutaneous” OR “sinus”) AND (“microb*” OR “biofilm*” OR “bacteria” OR “microorg*” OR “microflora” OR “dysbio*” OR “bacterial communit*” OR “plaque”)).

The full Boolean query and detailed search strategy are presented in Table A1. This initial retrieval yielded 1759 publications. After and within the database, the following filters were applied: a publication date between 2006 and 2025 (both years inclusive), only documents classified as articles and reviews, and publications in the English language. The total number of documents retrieved for screening was 1251, comprising 1081 articles and 170 reviews.

Prior to screening, duplicate publications (*n* = 5) and retracted articles (*n* = 2) were removed, as well as 9 documents published in 2026, despite having applied the publication date filter for 2006–2025.

The inclusion criteria focused on the following domains: publications on oral hygiene products with microbiome-modulating agents or antimicrobial activity and their effects on dental biofilm and the oral microbiome, including oral and systemic side effects, as well as all study designs within the scientific evidence pyramid, including animal research. Based on the search criteria, articles whose main topics focused on the microbiota and biofilm of the gut, nasopharynx, respiratory tract, skin, or sinuses were excluded.

The exclusion criteria were articles whose main topic focused on oral care products without antimicrobial activity (e.g., anti-tartar or whitening agents), materials with antimicrobial properties, and in-office dental procedures involving antimicrobial products.

**Figure 10:**
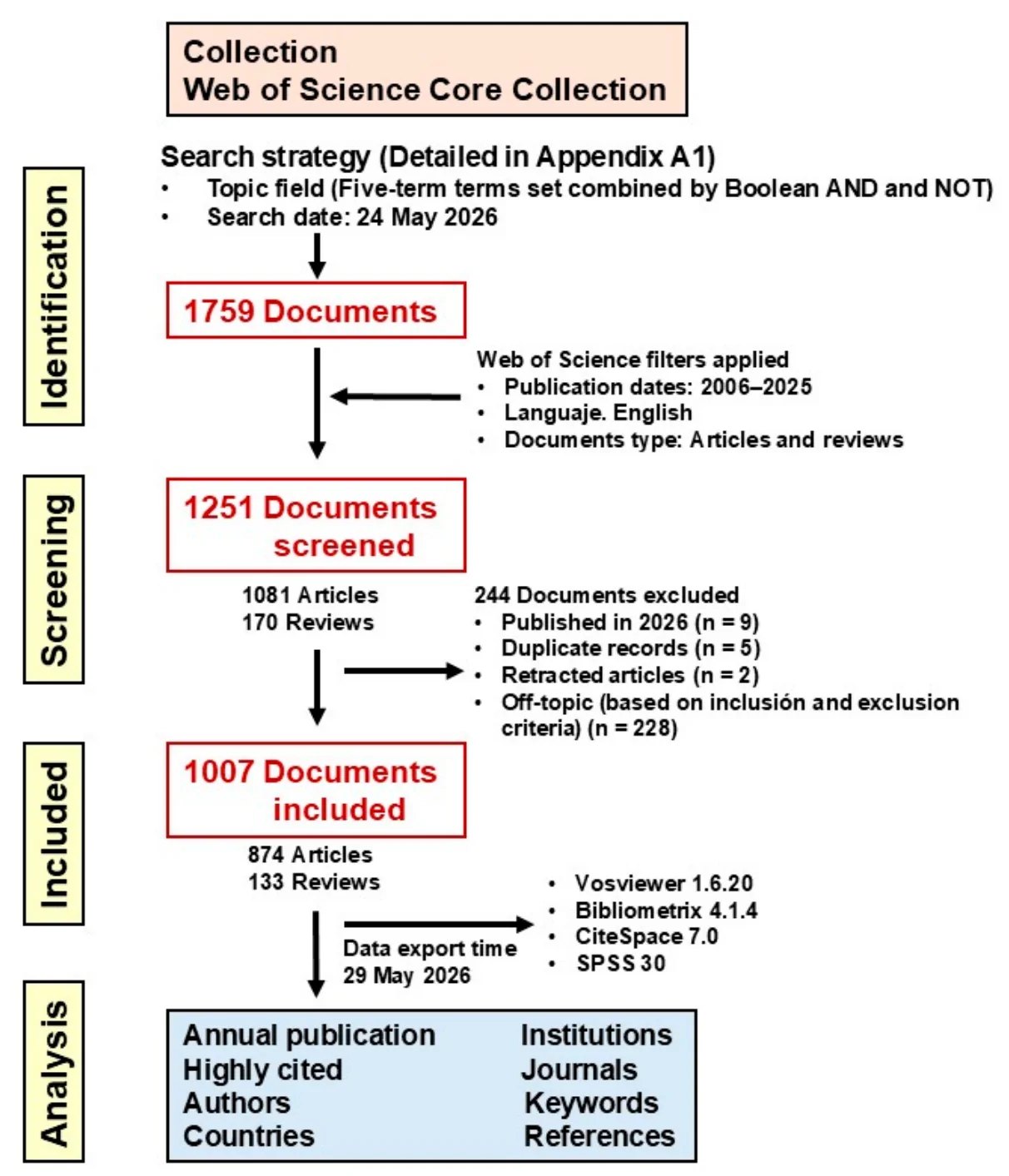
Flowchart of the bibliometric analysis process.

Two researchers (A.B-G. and P.B.) independently and blindly screened titles, abstracts, and, where necessary, full texts to select only those documents that aligned with the predefined inclusion criteria. Any discrepancies or doubts during the screening process were resolved through discussion until a consensus was reached between both investigators. Following the screening process, a total of 228 documents were excluded. Ultimately, 1007 relevant papers (874 articles and 133 reviews) were identified and selected for the final bibliometric analysis; the complete selection workflow is illustrated in Figure 10. On 29 May 2026, the unique Web of Science accession numbers (WoS IDs) were utilised to retrieve and export the final dataset of 1007 documents. The WoSCC database provided enriched metadata for these records, including complete cited reference lists, citation metrics, comprehensive author information, institutional affiliations, geographic data, journal details, and keywords. This comprehensive metadata was extracted in plain text, BibTeX and Microsoft Excel (.xlsx)formats. To ensure full reproducibility, the specific input files utilised across the software platforms are provided as Supplementary Files S1 and S2, whilst the complete dataset is made available in Microsoft Excel format as Supplementary File S3.

### 4.2. Data Analysis

Visual analysis and knowledge maps were generated using VOSviewer (version 1.6.20, Centre for Science and Technology Studies, Leiden University, Leiden, The Netherlands), CiteSpace (version 7.0, Drexel University, Philadelphia, PA, USA), and the R package ‘bibliometrix’ (version 4.1.4). IBM SPSS Statistics (version 30) was used to generate Figure 1A,B.

To ensure analytical consistency and avoid bias, identical criteria for data cleaning and term consolidation (grouping singular and plural forms, synonyms, and abbreviations) were applied across the three platforms. This process was carried out on author keywords and was done manually and agreed upon by two researchers (A.B-G. and P.B.) following initial exploratory analyses. The specific configurations and thesaurus files used to unify these terms in each programme (VOSviewer, CiteSpace, and the R ‘bibliometrix’ package) are provided as Supplementary Files S4–S6 (in plain text format). A manual process of author and institution disambiguation was carried out to group all variants. This process was performed by two researchers (P.B. and P.V.) using an Excel database, calculating the number of documents and citations for each author and institution (Table 2 and Table 4).

VOSviewer was utilised to model social collaboration networks (authors, countries, and institutions) applying a minimum productivity threshold of three documents (≥3) per node. Conceptual structures were evaluated through author keywords co-occurrence analysis using an inclusion threshold of six or more occurrences (≥6), which successfully filtered exactly 89 keywords out of 1773 total unique terms. Network distances and cluster boundaries were computed using the default association strength normalisation method, partitioning the keyword landscape into 7 distinct thematic clusters.

CiteSpace was used to determine the top 25 references with the strongest citation bursts to operationalize active research fronts up to 2025. In addition, it was used to identify a reference co-citation network. It was configured with time slicing from 2006 to 2025 (Slice Length = 1); the selection criteria were top 50 per slice, and the network topology was optimised via the Pathfinder pruning algorithm. The resulting structural framework generated 3725 nodes and 11,443 links (density = 0.0016; largest connected component = 69%), validating network robustness with an overall modularity (Q) score of 0.9407 (>0.3 indicates significant structure), and a weighted mean silhouette (S) score of 0.9562 (>0.7 indicates high confidence). Thematic clusters were automatically named using Log-Likelihood Ratio (LLR) algorithmic extraction from citing titles. Original node designations and algorithmic cluster labels were strictly preserved without manual intervention.

The R package ‘bibliometrix’ was utilised to evaluate the corresponding authors’ country distributions, and core specialised scientific journals were identified and ranked by applying Bradford’s Law of Scattering, mapping their annual cumulative publication output over time. The trend topic plot was constructed using the top three most frequent author keywords within each specific year. Finally, longitudinal thematic evolution pathways and strategic quadrant clustering were constructed. This analysis used a co-word nexus approach divided into four distinct chronological sub-periods—2006–2014 (*n* = 260 documents), 2015–2019 (*n* = 261 documents), 2020–2022 (*n* = 235 documents), and 2023–2025 (*n* = 251 documents)—to map thematic transitions in an alluvial diagram alongside strategic quadrant maps (Motor, Niche, Basic, and Emerging/Declining themes) categorised according to keyword centrality (x-axis) and density (y-axis). The four chronological sub-periods used for the strategic diagrams were calculated by balancing the dataset to ensure a similar number of publications per period.

The exact configurations, algorithmic parameters, thresholds, and normalisation procedures utilised across all three analysis platforms are comprehensively summarised in Supplementary Table S2.

## 5. Conclusions

Considering the limitations of this study, the most significant findings of this bibliometric analysis highlight a transition that seems consolidated within dentistry: research has evolved from a traditional, non-selective antimicrobial approach, focused on eliminating specific pathogens such as *Streptococcus mutans* and *Porphyromonas gingivalis*, towards strategies directed at the biological modulation of the oral microbiome. The oral microbiome forms a relatively recent, high-volume, and highly influential body of work that is still in an active phase of investigation; it is not an isolated ecosystem and can therefore significantly influence systemic health and disease. Classic antiseptics like CHX maintain a fundamental presence backed by a substantial volume of research, but research increasingly examines the potential systemic implications of broad-spectrum oral antisepsis, particularly effects on nitrate-reducing oral bacteria and nitric oxide pathways.

Furthermore, there is a surge in the literature seeking alternative oral antimicrobial formulations aimed at the ecological management of oral health without causing dysbiosis. This includes a better understanding of familiar formulations such as essential oils, e.g., tea tree oil and cymenol, and natural therapies like herbal extracts, but it also targets newer strategies such as microbiome-based therapies, including probiotics, postbiotics, or phage therapy, or nanoparticles, among others.

While these bibliometric trends reflect academic interest, their application to clinical practice will require practitioners to assess whether the benefits outweigh the potential oral and systemic risks. Resolving this uncertainty requires high-quality research that translates into clinical recommendations and guidelines, which may need to be tailored to each patient’s specific oral and systemic condition, potentially serving as a valuable tool for both practitioners and public dental services.

## Figures and Tables

**Table 1:** The 10 most cited documents about antimicrobial oral care products on dental biofilm ranked according to the highest citations. 2006–2025.

Rank	Documents	Cites WoSCC	Citation/Year (Rank)	Thematic Field
1	Govoni, M.; Jansson, E.A.; Weitzberg, E.; Lundberg, J.O. The increase in plasma nitrite after a dietary nitrate load is markedly attenuated by an antibacterial mouthwash. Nitric Oxide 2008, 19, 333–337.	511	28.39 (5)	Systemic disorders/antibacterial mouthwash
2	James, P.; Worthington, H.V.; Parnell, C.; Harding, M.; Lamont, T.; Cheung, A.; Whelton, H.; Riley, P. Chlorhexidine mouthrinse as an adjunctive treatment for gingival health. Cochrane Database Syst Rev 2017, 3, CD008676.	399	44.33 (3)	Effectiveness Chlorhexidine
3	Kapil, V.; Haydar, S.M.; Pearl, V.; Lundberg, J.O.; Weitzberg, E.; Ahluwalia, A. Physiological role for nitrate-reducing oral bacteria in blood pressure control. Free Radic Biol Med 2013, 55, 93–100.	321	24.69 (7)	Systemic disorders/antibacterial mouthwash
4	Petti, S.; Scully, C. Polyphenols, oral health and disease: A review. J Dent 2009, 37, 413–423.	277	16.29 (16)	Polyphenols
5	Brookes ZLS, Bescos R, Belfield LA, Ali K, Roberts A. Current uses of chlorhexidine for management of oral disease: a narrative review. J Dent 2020;103:103497	273	45.50 (2)	Chlorhexidine/Oral microbiome
6	Van Strydonck DA, Slot DE, Van der Velden U, Van der Weijden F. Effect of a chlorhexidine mouthrinse on plaque, gingival inflammation and staining in gingivitis patients: a systematic review. J Clin Periodontol 2012;39:1042–55.	255	18.21 (12)	Effectiveness Chlorhexidine
7	Gunsolley JC. A meta-analysis of six-month studies of antiplaque and antigingivitis agents. J Am Dent Assoc 2006;137:1649–57.	236	11.80 (35)	Several antimicrobial agents
8	Burton JP, Chilcott CN, Moore CJ, Speiser G, Tagg JR. A preliminary study of the effect of probiotic Streptococcus salivarius K12 on oral malodour parameters. J Appl Microbiol 2006;100:754–64.	204	10.20 (44)	Probiotic
9	Petersson J, Carlström M, Schreiber O, Phillipson M, Christoffersson G, Jägare A, Roos S, Jansson EA, Persson AE, Lundberg JO, Holm L. Gastroprotective and blood pressure lowering effects of dietary nitrate are abolished by an antiseptic mouthwash. Free Radic Biol Med 2009;46:1068–75.	202	11.88 (34)	Systemic disorders /antibacterial mouthwash
10	Rajasekaran JJ, Krishnamurthy HK, Bosco J, Jayaraman V, Krishna K, Wang T, Bei K. Oral Microbiome: A Review of Its Impact on Oral and Systemic Health. Microorganisms 2024;12:1797.	198	99.00 (1)	Oral microbiome/systemic health

WoSCC: Web of Science Core Collection. Citation/year = cites WoSCC/(2026 − year of publication).

**Table 2:** Top ten most productive authors ranked by number of documents.

Rank	Author	Institution (Country) **	Documents	Citations	h-Index *
1	Sreenivasan PK	Colgate-Palmolive Technology Center (United States)	28	459	29
2	Van der Weijden GA	Vrije Universiteit Amsterdam (Netherlands)	25	873	61
3	Slot DE	Vrije Universiteit Amsterdam (Netherlands)	15	598	39
4	Herrera D	University Complutense, Madrid (Spain)	13	499	56
5	Busscher HJ	University of Groningen (Netherlands)	13	273	96
6	Al-Ahmad A	University of Freiburg (Germany)	12	307	37
7	Van der Mei HC	University of Groningen (Netherlands)	12	239	79
8	Sanz M	Complutense University, Madrid (Spain)	11	297	71
9	Hannig M	Technical University of Dresden (Germany)	11	273	46
10	Serrano J	University Complutense, Madrid (Spain)	10	429	24

* Overall career h-index values were retrieved from the Web of Science Core Collection in May 2026. ** When an author is assigned to several institutions, the one with the highest frequency has been selected.

**Table 3:** Top ten most productive countries.

Rank	Country	Documents (%)	Citations	CPD	TLS
1	United States	187 (18.57)	4621	24.71	129
2	India	138 (13.70)	1392	10.08	41
3	Brazil	78 (7.77)	1386	17.77	29
4	China	69 (6.85)	1107	16.04	40
5	United Kingdom	65 (6.45)	2662	40.95	69
6	Netherlands	56 (5.56)	1651	29.48	34
7	Italy	55 (5.46)	1338	24.33	47
8	Germany	53 (5.26)	1220	23.02	38
9	Iran	52 (5.16)	591	11.37	7
10	Saudi Arabia	44 (4.37)	712	16.18	43

A total of 79 countries contributed to the 1007 documents. Document counts were calculated using full counting based on all author affiliations. CPD: Citations per document. TLS: Total link strength (≥3 co-occurrences).

**Table 4:** Top ten most productive Institutions.

Rank	Institutions	Documents	Citations	CPD
1	University of Amsterdam (Netherlands)	22	821	37.32
2	University of São Paulo (Brazil)	22	279	12.68
3	Vrije Universiteit Amsterdam (Netherlands)	19	524	27.58
4	Saveetha University (India)	18	41	2.16
5	Colgate-Palmolive (United States)	17	274	16.12
6	Procter & Gamble (United States)	15	198	13.20
7	Sichuan University (China)	15	375	25.00
8	University of Groningen (Netherlands)	15	400	26.67
9	University of Freiburg (Germany)	14	404	28.86
10	University of Hong Kong (China)	14	275	16.64

CPD: Citations per document.

**Table 5:** Top 16 journals of Core zone (zone 1) by Bradford’s Law.

Rank	Journal	Documents	Citations	IF (2025)	Quartile (Percentile)	Category
1	*International Journal of Dental Hygiene*	48	573	1.2	Q4 (25.2)	Dentistry
2	*Clinical Oral Investigations*	34	546	3.6	Q1 (81.5)	Dentistry
3	*BMC Oral Health*	32	414	3.8	Q1 (83.9)	Dentistry
4	*Journal of Dentistry*	25	1356	5.8	Q1 (94.2)	Dentistry
5	*Journal of Clinical Periodontology*	24	1103	8.3	Q1 (98.5)	Dentistry
6	*Oral Health & Preventive Dentistry*	22	224	1.7	Q3 (41.5)	Dentistry
7	*American Journal of Dentistry*	21	171	1.1	Q4 (24.5)	Dentistry
8	*Archives of Oral Biology*	19	472	2.3	Q2 (59.7)	Dentistry
9	*Journal of Periodontology*	18	635	4.5	Q1 (89.4)	Dentistry
10	*Cureus Journal of Medical Science*	18	130	-	-	No JCR 2025
11	*International Dental Journal*	17	279	5.2	Q1 (91.8)	Dentistry
12	*Journal of Clinical and Diagnostic Research*	15	147	0.2	Q4 (13.2)	Medicine, General & Internal
13	*Journal of the American Dental Association*	14	705	4.4	Q1 (88.8)	Dentistry
14	*Scientific Reports*	13	235	4.9	Q1 (85.4)	Multidisciplinary Sciences
15	Antibiotics *	12	117	5.5	Q1 (87.6)	Infectious Diseases
16	*Journal of Oral Microbiology*	12	175	6.7	Q1 (86.5)	Microbiology

* Also included in the Pharmacology and Pharmacy category (Q1, 84.4 percentile). Dentistry: Dentistry, Oral Surgery, and Medicine Journal distribution by Bradford’s Law in 3 zones: Core zone (zone 1), 16 journals, 344 articles; Intermediate zone (zone 2), 63 journals, 329 articles; and Peripheral zone (zone 3), 260 journals, 333 articles. The empirical distribution is consistent with Bradford’s Law.

**Table 6:** Top 20 keywords by frequency.

Rank	Keyword	Frequency	TLS
1	Dental plaque	293	752
2	Chlorhexidine	227	547
3	Mouthwash	224	580
4	Gingivitis	183	509
5	Toothpaste	105	284
6	Biofilm	98	203
7	Periodontitis	97	234
8	*Streptococcus mutans*	68	151
9	Oral Hygiene	56	128
10	Antimicrobial activity	54	113
11	Essential oil	52	134
12	Dental Caries	44	95
13	Cetylpiridinium chloride	41	127
14	Bacteria	40	124
15	Oral Health	37	91
16	Probiotics	32	71
17	Oral microbiome	30	86
18	Saliva	28	78
19	Fluoride	26	82
20	Triclosan	22	66

A total of 1773 authors’ keywords; 89 meet the threshold. TLS: Total link strength (≥6 occurrences). Quantifies how many other keywords co-appear in the same publications.

## Data Availability

Data are provided within the manuscript or Supplementary Information Files.

## Supplementary Materials

The following supporting information can be downloaded at: https://www.mdpi.com/article/10.3390/antibiotics15090839/s1, File S1: data in plain text of the 1007 documents; File S2: data in bibtex of the 1007 documents; File S3: Raw data sheet of the 1007 documents; File S4: Thesaurus. Disambiguation of keywords in VOSviewer; File S5: Thesaurus. Disambiguation of keywords in CiteSpace; File S6: Thesaurus. Disambiguation of keywords in R package ‘bibliometrix’; Table S1: Reference co-citation network and summary data for the resulting clusters (ordered by size); Table S2: Methodological configuration of the bibliometric analysis platforms.

## Abbreviations

The following abbreviation is used in this manuscript:

CHX	Chlorhexidine

## Appendix A

**Table A1:** Search strategy in Web of Science Core Collection. 24 May 2026.

#1	(“mouthwash*” OR “mouth rinse*” OR “mouthrinse*” OR “rinse*” OR “dentifrice*” OR “toothpaste*” OR “gel*” OR “spray*” OR “dental floss” OR “dental silk” OR “oral irrigator” OR “water flosser” OR “dental water jet”)
#2	(“anti plaque” OR “antibacterial agent*” OR “antimicrobial” OR “chemical agent*” OR “antiseptic*” OR “chlorhexidine” OR “hexetidine” OR “alexidine” OR “essential oil*” OR “quaternary ammonium” OR “cetylpyridinium” OR “benzalkonium” OR “povidone iodine” OR “hydrogen peroxide” OR “listerine” OR “amine fluoride*” OR “stannous” OR “zinc” OR “detergent*” OR “herbal” OR “triclosan” OR “peptide*” OR “probiotic*” OR “prebiotic*” OR “propolis” OR “Hydrogen” OR “delmopinol” OR “curcum*” OR “arginine” OR “*hyaluronic*” OR “peroxide”)
#3	(“oral microbiota” OR “oral microbiome” OR “oral biofilm*” OR “oral bacteria*” OR “oral microorg*” OR “oral microflora” OR “oral flora” OR “oral dysbio*” OR “oral microenvironment*” OR “mouth microbiota*” OR “mouth microbiome” OR “mouth bacteria*” OR “salivary microbiota” OR “buccal microbiota” OR “dental microbiota” OR “dental microbiome” OR “dental plaque”)
#4	(“gut” OR “intestinal” OR “nasopharyngeal” OR “nasal” OR “respiratory” OR “bronchial” OR “tracheal” OR “skin” OR “cutaneous” OR “sinus”)
#5	(“microb*” OR “biofilm*” OR “bacteria” OR “microorg*” OR “microflora” OR “dysbio*” OR “bacterial communit*” OR “plaque”)
#6 = #1 AND #2 AND #3	((“mouthwash*” OR “mouth rinse*” OR “mouthrinse*” OR “rinse*” OR “dentifrice*” OR “toothpaste*” OR “gel*” OR “spray*” OR “dental floss” OR “dental silk” OR “oral irrigator” OR “water flosser” OR “dental water jet”) AND (“anti plaque” OR “antibacterial agent*” OR “antimicrobial” OR “chemical agent*” OR “antiseptic*” OR “chlorhexidine” OR “hexetidine” OR “alexidine” OR “essential oil*” OR “quaternary ammonium” OR “cetylpyridinium” OR “benzalkonium” OR “povidone iodine” OR “hydrogen peroxide” OR “listerine” OR “amine fluoride*” OR “stannous” OR “zinc” OR “detergent*” OR “herbal” OR “triclosan” OR “peptide*” OR “probiotic*” OR “prebiotic*” OR “propolis” OR “Hydrogen” OR “delmopinol” OR “curcum*” OR “arginine” OR “*hyaluronic*” OR “peroxide”) AND (“oral microbiota” OR “oral microbiome” OR “oral biofilm*” OR “oral bacteria*” OR “oral microorg*” OR “oral microflora” OR “oral flora” OR “oral dysbio*” OR “oral microenvironment*” OR “mouth microbiota*” OR “mouth microbiome” OR “mouth bacteria*” OR “salivary microbiota” OR “buccal microbiota” OR “dental microbiota” OR “dental microbiome” OR “dental plaque”))
#7 = #4 AND #5	((“gut” OR “intestinal” OR “nasopharyngeal” OR “nasal” OR “respiratory” OR “bronchial” OR “tracheal” OR “skin” OR “cutaneous” OR “sinus”) AND (“microb*” OR “biofilm*” OR “bacteria” OR “microorg*” OR “microflora” OR “dysbio*” OR “bacterial communit*” OR “plaque”))
#8 = #6 NOT #7	((“mouthwash*” OR “mouth rinse*” OR “mouthrinse*” OR “rinse*” OR “dentifrice*” OR “toothpaste*” OR “gel*” OR “spray*” OR “dental floss” OR “dental silk” OR “oral irrigator” OR “water flosser” OR “dental water jet”) AND (“anti plaque” OR “antibacterial agent*” OR “antimicrobial” OR “chemical agent*” OR “antiseptic*” OR “chlorhexidine” OR “hexamidine” OR “alexidine” OR “essential oil*” OR “quaternary ammonium” OR “cetylpyridinium” OR “benzalkonium” OR “povidone iodine” OR “hydrogen peroxide” OR “listerine” OR “amine fluoride*” OR “stannous” OR “zinc” OR “detergent*” OR “herbal” OR “triclosan” OR “peptide*” OR “probiotic*” OR “prebiotic*” OR “propolis” OR “Hydrogen” OR “delmopinol” OR “curcum*” OR “arginine” OR “*hyaluronic*” OR “peroxide”) AND (“oral microbiota” OR “oral microbiome” OR “oral biofilm*” OR “oral bacteria*” OR “oral microorg*” OR “oral microflora” OR “oral flora” OR “oral dysbio*” OR “oral microenvironment*” OR “mouth microbiota*” OR “mouth microbiome” OR “mouth bacteria*” OR “salivary microbiota” OR “buccal microbiota” OR “dental microbiota” OR “dental microbiome” OR “dental plaque”)) NOT ((“gut” OR “intestinal” OR “nasopharyngeal” OR “nasal” OR “respiratory” OR “bronchial” OR “tracheal” OR “skin” OR “cutaneous” OR “sinus”) AND (“microb*” OR “biofilm*” OR “bacteria” OR “microorg*” OR “microflora” OR “dysbio*” OR “bacterial communit*” OR “plaque”))

## References

[1] Nayak S.C., Latha P.B., Kandanattu B., Pympallil U., Kumar A., Banga H.K. (2025). The Oral Microbiome and Systemic Health: Bridging the Gap Between Dentistry and Medicine. Cureus.

[2] Deo P., Deshmukh R. (2019). Oral microbiome: Unveiling the fundamentals. J. Oral Maxillofac. Pathol..

[3] Kilian M., Chapple I.L.C., Hannig M., Marsh P.D., Meuric V., Pedersen A.M., Tonetti M.S., Wade W.G., Zaura E. (2016). The oral microbiome—An update for oral healthcare professionals. Br. Dent. J..

[4] Pitts N.B., Zero D.T., Marsh P.D., Ekstrand K., Weintraub J.A., Ramos-Gomez F., Tagami J., Twetman S., Tsakos G., Ismail A. (2017). Dental caries. Nat. Rev. Dis. Primers.

[5] Mehrnia N., Van Dyke T.E. (2026). Microbial dysbiosis and immune dysregulation in periodontitis and peri-implantitis. Front. Cell. Infect. Microbiol..

[6] Guo Z.L., Cui M.W., Dong Y.L., Wang S. (2026). The oral microbiome as a regulatory hub for systemic health: A systematic review of mechanistic links and clinical implications. J. Oral Microbiol..

[7] Arbildo-Vega H.I., Cruzado-Oliva F.H., Coronel-Zubiate F.T., Meza-Málaga J.M., Luján-Valencia S.A., Luján-Urviola E., Echevarria-Goche A., Farje-Gallardo C.A., Castillo-Cornock T.B., Serquen-Olano K. (2024). Periodontal disease and cardiovascular disease: Umbrella review. BMC Oral Health.

[8] Wu C.Z., Yuan Y.H., Liu H.H., Li G.R., Zhang B.X., Chen W.Q., An Z.J., Chen S.Y., Wu Y.Z., Han B. (2020). Epidemiologic relationship between periodontitis and type 2 diabetes mellitus. BMC Oral Health.

[9] Lv W., Hu H., Huang Y., Yang J., Li Y., He J., Wang K., Liu Y., Wang Q. (2026). Microbial mechanisms and therapeutic interventions in the periodontitis-inflammatory bowel disease axis: A comprehensive review. J. Oral Microbiol..

[10] Weber C., Dilthey A., Finzer P. (2023). The role of microbiome-host interactions in the development of Alzheimer’s disease. Front. Cell. Infect. Microbiol..

[11] du Teil Espina M., Gabarrini G., Harmsen H.J.M., Westra J., van Winkelhoff A.J., van Dijl J.M. (2019). Talk to your gut: The oral-gut microbiome axis and its immunomodulatory role in the etiology of rheumatoid arthritis. FEMS Microbiol. Rev..

[12] Tortora S.C., Agurto M.G., Martello L.A. (2023). The oral-gut-circulatory axis: From homeostasis to colon cancer. Front. Cell. Infect. Microbiol..

[13] Gupta A., Saleena L.M., Kannan P., Shivachandran A. (2024). The impact of oral diseases on respiratory health and the influence of respiratory infections on the oral microbiome. J. Dent..

[14] García-Rios P., Rodríguez-Lozano F.J., González-Murcia R., Murcia L., Victoria-Montesinos D., García-Muñoz A.M. (2026). The role of oral dysbiosis in pregnancy complications: A systematic review and meta-analysis of preterm birth. J. Oral Microbiol..

[15] Van der Weijden F., Slot D.E. (2011). Oral hygiene in the prevention of periodontal diseases: The evidence. Periodontology 2000.

[16] Brookes Z.L.S., Belfield L.A., Ashworth A., Casas-Agustench P., Raja M., Pollard A.J., Bescos R. (2021). Effects of chlorhexidine mouthwash on the oral microbiome. J. Dent..

[17] Supranoto S.C., Slot D.E., Addy M., Van der Weijden G.A. (2015). The effect of chlorhexidine dentifrice or gel versus chlorhexidine mouthwash on plaque, gingivitis, bleeding and tooth discoloration: A systematic review. Int. J. Dent. Hyg..

[18] James P., Worthington H.V., Parnell C., Harding M., Lamont T., Cheung A., Whelton H., Riley P. (2017). Chlorhexidine mouthrinse as an adjunctive treatment for gingival health. Cochrane Database Syst. Rev..

[19] Pose-Otero F., Arredondo A., Parga A., Muras A., Gallas M., Otero-Casal P., Pose-Rodríguez J.M., Otero A. (2025). The Effect of Chlorhexidine Mouthwashes on the Microbiota Associated with Peri-Implantitis Lesions: A Pilot Study. Antibiotics.

[20] Van Strydonck D.A., Slot D.E., Van der Velden U., Van der Weijden F. (2012). Effect of a chlorhexidine mouthrinse on plaque, gingival inflammation and staining in gingivitis patients: A systematic review. J. Clin. Periodontol..

[21] Kotsailidi E.A., Kalogirou E.M., Michelogiannakis D., Vlachodimitropoulos D., Tosios K.I. (2020). Hypersensitivity reaction of the gingiva to chlorhexidine: Case report and literature review. Oral Surg. Oral Med. Oral Pathol. Oral Radiol..

[22] Figuero E., Herrera D., Tobías A., Serrano J., Roldán S., Echeverría J.J. (2019). Efficacy of adjunctive anti-plaque chemical agents in gingivitis management: A systematic review and network meta-analysis. J. Clin. Periodontol..

[23] Jayanandan M., Veeraraghavan V.P., Govindarajan S., Mariyappa Subramani S. (2026). Development of oral dysbiosis following use of antimicrobial mouthwashes: A systematic review. Odontology.

[24] Alrashdan M.S., Leao J.C., Doble A., McCullough M., Porter S. (2023). The Effects of Antimicrobial Mouthwashes on Systemic Disease: What Is the Evidence?. Int. Dent. J..

[25] Angjelova A., Jovanova E., Polizzi A., Leonardi R., Isola G. (2025). Effects of Antiseptic Formulations on Oral Microbiota and Related Systemic Diseases: A Scoping Review. Antibiotics.

[26] Boulares A., Jdidi H., Bragazzi N.L. (2025). Impact of Mouthwash-Induced Oral Microbiome Disruption on Alzheimer’s Disease Risk: A Perspective Review. Int. Dent. J..

[27] Toonen L.S.J., Van Swaaij B.W.M., Vagevuur M.L., Van der Weijden F.G.A., Timmerman M.F., Slot D.E. (2026). The Effect of Chlorhexidine Mouthwash on Blood Pressure: A Systematic Review and Meta-Analysis. Int. J. Dent. Hyg..

[28] Li S., Huang X., McNeil R., Malmstrom H., Ren Y. (2026). Systemic health implications of dental prescribing in general practices. Quintessence Int..

[29] Duane B., Yap T., Neelakantan P., Anthonappa R., Bescos R., McGrath C., McCullough M., Brookes Z. (2023). Mouthwashes: Alternatives and Future Directions. Int. Dent. J..

[30] Liao G., Wu J., Peng X., Li Y., Tang L., Xu X., Deng D., Zhou X. (2020). Visualized analysis of trends and hotspots in global oral microbiome research: A bibliometric study. MedComm.

[31] Li Z., Fu R., Huang X., Wen X., Zhang L. (2023). A decade of progress: Bibliometric analysis of trends and hotspots in oral microbiome research (2013–2022). Front. Cell. Infect. Microbiol..

[32] Gu Z., Liu Y. (2024). A bibliometric and visualized in oral microbiota and cancer research from 2013 to 2022. Discov. Oncol..

[33] Pervaiz U., Nabeel P., Zhao R., Zhao Z., Zhang Y., Wang D. (2025). Global Research Trends on the Links Between the Oral Microbiome and Cancer From 2014 to 2024: A Visualization Analysis. J. Oral Pathol. Med..

[34] Espinoza-Carhuancho F., Huaman-De la Cruz M., Lozano-Castro F., Mauricio-Vilchez C., Munive-Degregori A., Mayta-Tovalino F. (2025). Global Patterns, Impact, and Networking in Mouthwash and Cancer: A Scientometric Analysis. J. Contemp. Dent. Pract..

[35] Wang X., Ma Y., Yang Y., Dong Q. (2026). Mapping the Research Landscape and Evolutionary Trends of the Oral Microbiome in Periodontitis. Apmis.

[36] Dagli N., Haque M., Kumar S. (2024). Mapping the Landscape of Clinical Trials on Oral Biofilm: A Bibliometric Analysis. Bangladesh J. Med. Sci..

[37] Farias da Cruz M., Baraúna Magno M., Alves Jural L., Pimentel T.C., Masterson Tavares Pereira Ferreira D., Almeida Esmerino E., Luis Paiva Anciens Ramos G., Vicente Gomila J., Cristina Silva M., Cruz A.G.D. (2022). Probiotics and dairy products in dentistry: A bibliometric and critical review of randomized clinical trials. Food Res. Int..

[38] Zhou F., Mu X., Li Z., Guo M., Wang J., Long P., Wan Y., Yuan T., Lv Y. (2023). Characteristics of Chinese herbal medicine mouthwash clinical studies: A bibliometric and content analysis. J. Ethnopharmacol..

[39] Ateş Yıldırım E., Meracı Yıldıran B. (2026). A Bibliometric Analysis of Chlorhexidine In Dentistry. Selcuk. Dent. J..

[40] Govoni M., Jansson E.A., Weitzberg E., Lundberg J.O. (2008). The increase in plasma nitrite after a dietary nitrate load is markedly attenuated by an antibacterial mouthwash. Nitric Oxide.

[41] Brookes Z.L.S., Bescos R., Belfield L.A., Ali K., Roberts A. (2020). Current uses of chlorhexidine for management of oral disease: A narrative review. J. Dent..

[42] Bescos R., Ashworth A., Clarke C., Brookes Z.L., Belfield L., Rodiles A., Casas-Agustench P., Farnham G., Liddle L., Burleigh M. (2020). Effects of Chlorhexidine mouthwash on the oral microbiome. Sci. Rep..

[43] Rogers G., Szomszor M., Adams J. (2020). Sample size in bibliometric analysis. Scientometrics.

[44] Bornmann L., Haunschild R., Mutz J. (2021). Growth rates of modern science: A latent piecewise growth curve approach to model publication numbers from established and emerging countries. Humanit. Soc. Sci. Commun..

[45] Sanz M., Herrera D., Kebschull M., Chapple I., Jepsen S., Beglundh T., Sculean A., Tonetti M.S., EFP Workshop Participants and Methodological Consultants (2020). Treatment of stage I–III periodontitis-The EFP S3 level clinical practice guideline. J. Clin. Periodontol..

[46] Donthu N., Kumar S., Mukherjee D., Pandey N., Lim W.M. (2021). How to conduct a bibliometric analysis: An overview and guidelines. J. Bus. Res..

[47] Huang C., Chen G., Lu B., Li C., Sun X. (2026). The Application of Magnetic Resonance Imaging in Dentistry: A Bibliometric Analysis. Int. Dent. J..

[48] Guo Y., Tong C., Wang T., Zhou L., Wang M. (2026). Research Trends in Autogenous Dentin Graft: A Bibliometric and Cluster Analysis (2006–2025). Int. Dent. J..

[49] Kapil V., Haydar S.M., Pearl V., Lundberg J.O., Weitzberg E., Ahluwalia A. (2013). Physiological role for nitrate-reducing oral bacteria in blood pressure control. Free Radic. Biol. Med..

[50] Rajasekaran J.J., Krishnamurthy H.K., Bosco J., Jayaraman V., Krishna K., Wang T., Bei K. (2024). Oral Microbiome: A Review of Its Impact on Oral and Systemic Health. Microorganisms.

[51] Guo C., Lu X. (2016). Selecting publication keywords for domain analysis in bibliometrics: A comparison of three methods. J. Informetr..

[52] Mohapatra S., Mohandas R. (2025). Comparative evaluation of the efficacy of cetylpyridinium chloride and essential oil mouthwashes in reducing plaque and gingivitis: A systematic review and meta-analysis. Evid. Based Dent..

[53] Zhang C., Liu B., Hu J., Zhao L., Zhao H. (2024). The Effect of Local Application of Tea Tree Oil Adjunctive to Daily Oral Maintenance and Nonsurgical Periodontal Treatment: A Systematic Review and Meta-Analysis of Randomised Controlled Studies. Oral Health Prev. Dent..

[54] Iniesta M., Vasconcelos V., Laciar F., Matesanz P., Sanz M., Herrera D. (2025). Impact of toothpaste use on the subgingival microbiome: A pilot randomized clinical trial. BMC Oral Health.

[55] Kong J.F., Phang H.C., Wan Kamal W.H.B., Ng Y., Mohamad S., Kee P.E., Liew K.B. (2026). Role of Probiotics in Oral Health: A Review From Microbial Balance to Clinical Applications. Curr. Pharm. Biotechnol..

[56] Pilloni A., Shirakata Y., Marini L., Božić D., Miron R.J., Rotundo R., Stavropoulos A., Sculean A. (2025). Hyaluronic acid: A novel approach in regenerative/reconstructive periodontal therapy?. Periodontology 2000.

[57] Scribante A., Pascadopoli M., Pellegrini M., Chekal K., Butera A. (2026). Efficacy of a Hyaluronic Acid-Based Gel Versus 0.20% Chlorhexidine in Nonsurgical Periodontal Therapy: A Single-Blind Randomized Clinical Trial. Oral Dis..

[58] Feria Q., Soegiharto I.R.S., Putri N.D.A., Hutapea Y., Takahashi N., Sulijaya B., Ayuningtyas D. (2026). The Efficacy of Sodium Hypochlorite in Combination with Hyaluronic Acid as an Adjunct to Non-Surgical Periodontal Treatment: A Systematic Review. Antibiotics.

[59] Cieplik F., Jakubovics N.S., Buchalla W., Maisch T., Hellwig E., Al-Ahmad A. (2019). Resistance Toward Chlorhexidine in Oral Bacteria—Is There Cause for Concern?. Front. Microbiol..

[60] Kulis E., Cvitkovic I., Pavlovic N., Kumric M., Rusic D., Bozic J. (2025). A Comprehensive Review of Antibiotic Resistance in the Oral Microbiota: Mechanisms, Drivers, and Emerging Therapeutic Strategies. Antibiotics.

[61] Poppolo Deus F., Ouanounou A. (2022). Chlorhexidine in Dentistry: Pharmacology, Uses, and Adverse Effects. Int. Dent. J..

[62] Khudhair H., Alhamdani A.S.H., Al-Tameemi A.M.H. (2026). Microbiology of dental decay and periodontal disease: A review. Wiad. Lek..

