# Supplementary material for: Research Trends in Antimicrobial Oral Hygiene Products, the Oral Microbiome, and Dental Biofilm: A Bibliometric Analysis (2006–2025)
Source: Antibiotics (Basel). 2026 Aug 29;15(9):839. doi: 10.3390/antibiotics15090839 (PMC13603143; doi:10.3390/antibiotics15090839)
Supplement: Supplementary file 1 [file antibiotics-15-00839-s001.zip › Supplementary Table S1.pdf]

Cluster Summary (41 Clusters by Size)

Selected	ClusterID	Size	Silhouette	Mean (Year)	Label (LSI)	Label (LLR)	Label (MI)
false	0	82	0.929	2018	medicinal plant extracts; melaleuca alternifolia oil; proper oral hygiene; herbal mouthwash; standard toothbrush	oral microbiome (2128.32, 1.0E-4); oral health (597.31, 1.0E-4); relative abundance (472, 1.0E-4); oral pathogen (463.74, 1.0E-4); systemic health (420.12, 1.0E-4)	gosau (7.01); sixty patient (7.01); potential risk
false	2	38	0.89	2008	peri-implant biofilm control; lmm mushroom; manual brushing; chx group; prepared chlorhexidine digluconate preparation | different oral health-care; peri-implant biofilm control; lmm mushroom; chx group; ms levels	chx varnish (193.06, 1.0E-4); ms level (193.06, 1.0E-4); unwanted side effect (160.84, 1.0E-4); manual brushing (160.84, 1.0E-4); chlorhexidine dentifrice (144.74, 1.0E-4)	cell measurement (0.12); common assumption (0.12);
false	3	31	0.923	2008	antiplaque effect; nct mouth rinses; natural antimicrobials; periodontal disease; oral biofilm model | antiplaque effect; chlorhexidine mouthwash; natural antimicrobials; periodontal disease; oral biofilm model	plaque vitality (228.8, 1.0E-4); scanning microscopy (182.1, 1.0E-4); essential oil-containing mouthrinse (171.28, 1.0E-4); gingival tissue (167.71, 1.0E-4); alveolar bone loss (167.71, 1.0E-4)	plaque biofilm (0.15); characteristic collection (
false	6	31	0.987	2009	saliva samples; volatile sulfur compounds; plaque control; significant differences; control group | saliva samples; volatile sulfur compounds; inorganic nitrate; plaque control;	blood pressure (384.47, 1.0E-4); antibacterial mouthwash	gosau (0.32); sixty patient (0.32); potential risk

					significant differences	(323.86, 1.0E-4); inorganic nitrate (290.34, 1.0E-4); plasma nitrite (265.06, 1.0E-4); nitrate load (265.06, 1.0E-4)	
false	8	31	0.929	2007	hexetidine product; noncontact brushing; cetylpyridinium chloride; antimicrobial mouthrinses; essential oil | hexetidine product; chx spray; chlorine dioxide mouthrinse; bond stiffness; control mouthrinse	chx spray (278.82, 1.0E-4); oral chemotherapeutics (248.27, 1.0E-4); residual biofilm (165.37, 1.0E-4); mechanical cleaning (164.55, 1.0E-4); stress relaxation (149.44, 1.0E-4)	containing chlorine dioxide (0.11); chlorine dioxi
false	4	29	0.905	2005	clinical parameters; chx group; sustained killing; american dental association; salivary pellicles | hexetidine product; hop bract polyphenols; clinical parameters; triclosan-coated sutures; chx group	plaque-covered surface (378.62, 1.0E-4); plaque-free surface (226.84, 1.0E-4); triclosan-coated suture (167.94, 1.0E-4); antiplaque rinse (167.94, 1.0E-4); mu l (151.12, 1.0E-4)	contiguous supragingival plaque (0.1); contiguous
false	15	29	0.937	2012	hydro-alcohol solution; plaque parameters; clinical pgrm studies; oral bacteria; using ncr | cpc rinses; hydro-alcohol solution; plaque parameters; clinical pgrm studies; oral bacteria	oral microbiome (153.36, 1.0E-4); product use subject (147.42, 1.0E-4); alcohol-free mouthwash (125.62, 1.0E-4); assigned mouthrinse (116.04, 1.0E-4); tongue cleaner (111.27, 1.0E-4)	gosau (0.18); sixty patient (0.18); potential risk
false	1	28	0.908	2007	mechanical plaque control; anaerobic bacteria; human oral cavity; ecologic niches; baseline samples | antibacterial	microbead dentifrice (245.53, 1.0E-4); oral	supportive therapy (0.08); saburreum

					activity; zinc lactate; anaerobic bacteria; epifluorescence microscopy; sodium hexametaphosphate	streptococci (175.24, 1.0E-4); viable bacteria (140.15, 1.0E-4); mechanical plaque control (137.68, 1.0E-4); whole mixed saliva (122.6, 1.0E-4)	fusobacterium
false	13	28	0.99	2013	healthy subjects; high caries; total biofilm biomass; arginine-containing toothpaste; arginine-containing dentifrice | dead bacteria ratio; antibacterial activity; practical application; clinical cohort; new technology	buah merah (453.66, 1.0E-4); insoluble calcium compound (327.48, 1.0E-4); caries process (214.25, 1.0E-4); inhibition zone (210.77, 1.0E-4); acidic l-arginine (191.4, 1.0E-4)	2-day cultivation (0.13); day-old multispecies bio
false	9	27	0.917	2014	inflammatory condition; oral health barleria prionitis; herbal extracts; periodontal disease; antimicrobial resistance | orthodontic bracket objectiveto; aloe vera; baseline plaque scores; inflammatory condition; oral health barleria prionitis	chlorhexidine gluconate mouthwash (269.31, 1.0E-4); aloe vera mouthwash (243.19, 1.0E-4); gingival health (210.82, 1.0E-4); herbal product (208.37, 1.0E-4); statistical analysis (161.88, 1.0E-4)	gosau (0.08); sixty patient (0.08); potential risk
false	10	22	0.96	2012	antibacterial activity; cpc rinses; daily plaque control; oral microbiota; selective inhibition | cpc rinses; oral microbiota; human population; cetylpyridinium chloride; toothbrushing rinsing	dental plaque composition (113.42, 1.0E-4); multi-species biofilm (94.5, 1.0E-4); fulvic acid (94.5, 1.0E-4); oral salivary microorganism (75.58, 1.0E-4);	chloride-based mouthwash (0.05); based mouthwashe

						selective inhibition (56.67, 1.0E-4)	
false	21	22	0.945	2011	natural compounds; lmm mushroom; antibacterial effects; oral hygiene measures; triphala mouth rinse | herbal mouthwash; aggregatibacter actinomycetemcomitans; different concentrations; pomegranate-containing mouthwash; anti discoloration system	pomegranate-containing mouthwash (185.44, 1.0E-4); anti-plaque effect (160.67, 1.0E-4); antiplaque activity (110.69, 1.0E-4); dental staining (109.4, 1.0E-4); anti discoloration system (109.4, 1.0E-4)	group i placebo mouthwash (0.08); group iii chlorh
false	5	21	0.966	2005	lactic acid; targeted killing; mutans-specific delivery; mutans biofilm; non-brushing period | lactic acid; targeted killing; mutans-specific delivery; mutans biofilm; antimicrobial peptide domain	streptococcus mutan (131.79, 1.0E-4); water-soluble polymer (93.33, 1.0E-4); non-brushing period (82.94, 1.0E-4); mutans biofilm (72.57, 1.0E-4); csp sequence (62.19, 1.0E-4)	dynamic biofilmbackground (0.03); daily toothbrush
false	18	21	0.973	2011	alcohol-based essential oils; overall pi; placebo solution; negative control; fda subcommittee | zinc lactate; alcohol-free eo; alcohol-based essential oils; overall pi; placebo solution	terminalia chebula mouth rinse (114.51, 1.0E-4); pomegranate mouthrinse (85.84, 1.0E-4); adverse effect (69.87, 1.0E-4); plaque regrowth (57.42, 1.0E-4); mouth rinse (57.27, 1.0E-4)	3-day supragingival plaque regrowth (0.05); gosau
false	11	19	0.946	2008	control mouthrinse; control fluoride mouthrinse; chlorhexidine mouthrinse; antimicrobial activity; prebrushing mouthwashes | available mouth rinse; early dental plaque regrowth; supragingival plaque; antimicrobial effects; anaerobic bacteria	anaerobic bacteria (208.49, 1.0E-4); fluoride mouth rinse (197.97, 1.0E-4); prebrushing mouthwashe (158.3, 1.0E-4);	other product (0.04); beneficial long-term effect

						microbiologic analyses (98.85, 1.0E-4); assigned mouth rinse (98.85, 1.0E-4)	
false	12	19	0.975	2009	weak antibacterial effects; bacterial morphology; enterococcus faecalis; herbal mouthwash; antibacterial effects | weak antibacterial effects; bacterial morphology; antibacterial effects; salviathymol n; plaque formation	salvadora persica l (182.24, 1.0E-4); hoggar miswak (177.38, 1.0E-4); aqueous extract (161.34, 1.0E-4); degrees c (141.03, 1.0E-4); physicochemical parameter (118.15, 1.0E-4)	tooth cleaning (0.04); marked antibacterial effect
false	17	19	0.967	2012	herbal mouthwash; saliva samples; chlorhexidine mouthwash; antimicrobial effect; oral infection | herbal toothpaste; non-herbal toothpaste; non-herbal toothpaste usage; herbal mouthwash; saliva samples	herbal toothpaste (131.75, 1.0E-4); non-herbal toothpaste (131.75, 1.0E-4); brazilian ethanol (109.74, 1.0E-4); alternative mouthwash (87.75, 1.0E-4); non-herbal toothpaste usage (65.79, 1.0E-4)	broth microdilution test method (0.02); vitro stud
false	33	19	0.99	2013	outcome measures; mean treatment effects; cpc-containing mouthrinse; developing good oral hygiene habits; lae-containing mouthrinse | eligibility criteria; outcome measures; plaque control; mean treatment effects; cpc-containing mouthrinse	mechanical method (200.56, 1.0E-4); mean percentage (177.42, 1.0E-4); containing mouthrinse (164.82, 1.0E-4); oral microbiome (157.78, 1.0E-4); healthy site (152.06, 1.0E-4)	chemical adjuvant (0.18); daily oral biofilm (0.18

false	14	18	0.947	2004	control mouthrinse; mucosal delivery; atomic absorption spectrometer; antigingivitis effect; containing essential oils | stannous fluoride dentifrice; sodium fluoride dentifrice; new chlorhexidine; eo rinses; theoretical risks	stannous fluoride dentifrice (118.19, 1.0E-4); laboratory studies (116.43, 1.0E-4); antiplaque efficacy (109.25, 1.0E-4); antiplaque antigingivitis effect (97, 1.0E-4); alcohol-free cetylpyridinium chloride mouthrinse (67.87, 1.0E-4)	containing asf (0.04); lowests plaque indice (0.04
false	28	18	0.987	2013	postoperative period; microbiological examinations; brazilian green propolis; containing eep-b; mean plaque scores | oral hygiene; oral health; systemic diseases; oral disease; containing eep-b	mouth washe (79.68, 1.0E-4); conventional chemical mean (79.68, 1.0E-4); oil swishing (79.68, 1.0E-4); evidence available (79.68, 1.0E-4); heart disease (79.68, 1.0E-4)	oral microbiome (0.02); physiological flora microo
false	31	18	0.997	2006	povidone-iodine group; octenidine dihydrochloride mouth rinse; occurrence rate; fifth day; chlorhexidine gluconate | occurrence rate; saliva samples; streptococcus mutans; lactobacillus species; chlorhexidine gluconate	occurrence rate (97.51, 1.0E-4); octenidine dihydrochloride (85.29, 1.0E-4); cariogenic bacteria (82.26, 1.0E-4); fifth day (73.08, 1.0E-4); fixed appliance (59.65, 1.0E-4)	oral microbiome (0.02); dental plaque (0.01); sixt
false	27	17	0.993	2009	containing mouthwash; meta-analysis alcohol; hydro-alcohol solution; normal brushing; plaque control	respectively p (239.77, 1.0E-4); cetylpyridinium chloride-containing mouthrinse (110.11, 1.0E-4);	gosau (0.11); sixty patient (0.11); potential risk

						oral microbiome (102.68, 1.0E-4); essential oil mouthwash (95.69, 1.0E-4); plaque regrowth (87.51, 1.0E-4)	
false	22	15	0.976	2009	simulated supragingival plaques; eubacterial dna; oral bacteria; herbal formulation; viable counting | drip flow reactors; herbal mouthwash; triclosan dentifrice; gingival status; conventional modes	dense plaque (88.18, 1.0E-4); viability reduction (66.11, 1.0E-4); viable counting (64.75, 1.0E-4); marked change (44.06, 1.0E-4); herbal formulations km (44.06, 1.0E-4)	herbal alternative (0.02); salivary streptococcus
false	23	15	0.976	2010	natural compounds; unsupervised oral hygiene; dental plaque; systematic review; propolis toothpaste | antibacterial activity; clove oil; using orthodontic appliances; oral infectious diseases; oral bacteria	clove oil (344.53, 1.0E-4); removable appliance (120.26, 1.0E-4); cleft lip (58.54, 1.0E-4); major compound (56.26, 1.0E-4); study clove oil (56.26, 1.0E-4)	oral microbiome (0.02); jadad scale (0.02); system
false	26	14	0.985	2008	plaque scores; antibacterial efficacy; minimum inhibitory concentration; antiplaque efficacy; antibacterial action | periodontal surgery; staining effects; stannous fluoride; mouth rinse; chlorhexidine digluconate	staining effect (77.92, 1.0E-4); test species (51.9, 1.0E-4); antibacterial action (42.34, 1.0E-4); dna-dna checkerboard (38.9, 1.0E-4); test chx rinse (38.9, 1.0E-4)	oral microbiome (0.02); dental plaque (0.01); oral
false	16	13	0.961	2004	daily use; positive control; mechanical plaque control; plaque control; oral health | plaque index; oral health; anti-inflammatory agents; anti-inflammatory effects; whole mouth	anti-inflammatory effect (72.53, 1.0E-4); dental plaque biofilm	oral microbiome (0.02); professional tooth cleanin

					plaque	(48.27, 1.0E-4); principal ligand (44.87, 1.0E-4); emerging therapeutic strategy (44.87, 1.0E-4); herbal-based toothpaste (44.87, 1.0E-4)	
false	29	13	0.984	2015	chronic periodontitis; non-surgical periodontal treatment; periodontal therapy; beta diversity; different change | fluoride toothpaste; bleeding index; chronic periodontitis; control group; test group	delivery system (92.17, 1.0E-4); inhibitory activity (92.17, 1.0E-4); guar-gum gel (92.17, 1.0E-4); agar-well diffusion assay (46.04, 1.0E-4); different change (46.04, 1.0E-4)	oral microbiome (0.02); using different type (0.02
false	32	12	0.997	2010	buccal mucosa; zinc lactate; antimicrobial effects; sodium hexametaphosphate; stannous fluoride | hortensis l; membrane permeability; mmp-9 activities; streptococcus mutans; epithelial cells	bacterial attachment (82.14, 1.0E-4); dental material (41.01, 1.0E-4); gingivalis biofilm (41.01, 1.0E-4); novel oligosaccharide nanomedicine oligog (41.01, 1.0E-4); oligog surface pretreatment (41.01, 1.0E-4)	oral microbiome (0.02); salvia fruticosa m (0.02);
false	38	12	0.988	2010	saline solution; mature biofilms; bacterial adherence; liquid phase; customary preparation | mature biofilms; oral health; adherent bacteria; control samples; edible oils	chitosan mouthwash (99.31, 1.0E-4); mature biofilm (66.11, 1.0E-4); antiplaque agent (51.45, 1.0E-4); adherent bacteria (50.2, 1.0E-4); liquid	oral microbiome (0.02); dental plaque (0.01); oral

						phase (42.39, 1.0E-4)	
false	7	11	0.979	2006	acid production; mutans streptococci; alkali production; preventive activity; oral diseases | oral diseases; vitro studies; fair evidence; preventive activity; preventive effect	alpha-mangostin (75.61, 1.0E-4); mutan (75.61, 1.0E-4); fair evidence (50.37, 1.0E-4); preventive effect (50.37, 1.0E-4); oral cancer (50.37, 1.0E-4)	oral microbiome (0.02); dental plaque (0.01); oral
false	20	11	0.996	2014	oral hygiene; oral microbiome; periodontal disease; dental caries; containing australian melaleuca alternifolia oil | oral hygiene; oral microbiome; sabdariffa calyx extracts; antimicrobial effect; antimicrobial activity	containing australian melaleuca alternifolia oil (73.4, 1.0E-4); polish propolis (73.4, 1.0E-4); medicinal plant (66.68, 1.0E-4); study group (56.8, 1.0E-4); sabdariffa extract (48.9, 1.0E-4)	oral microbiome (0.02); dental plaque (0.01); hibi
false	34	9	0.982	2014	antiseptic properties; weak effect; statistical analysis; synergistic effect; patient hygiene performance | odontological product; propolis-containing mouthwash; patient hygiene performance; dental plaque; microbial contamination	propolis (165.22, 1.0E-4); honey (126.92, 1.0E-4); aureus (50.63, 1.0E-4); synergistic effect (46.52, 1.0E-4); material (41.08, 1.0E-4)	oral microbiome (0.02); dental plaque (0.01); oral
false	36	9	0.999	2006	compositional stability; probiotic lactobacillus salivarius w24; lactobacillus salivarius w24; oral microbial communities; rrna gene segments | rrna gene segments; ma copolymer; microbial composition; supragingival plaque samples; rrna gene sequence analysis	microcosm (179.85, 1.0E-4); effect (79, 1.0E-4); community profile (51.14, 1.0E-4); sucrose (51.14, 1.0E-4); compositional stability (51.14, 1.0E-4)	oral microbiome (0.02); dental plaque (0.01); oral

false	39	8	1	2015	dental plaque levels; following stroke; oral health promotion; low-quality evidence; multi-component ohc intervention | low-quality evidence; stroke survivor; ohc intervention; ohc interventions; multi-component ohc intervention	participant (260.07, 1.0E-4); trial (193.83, 1.0E-4); ohc intervention (125.22, 1.0E-4); dental plaque level (97.08, 1.0E-4); data (79.6, 1.0E-4)	oral microbiome (0.02); provider (0.02); oral heal
false	50	8	0.997	2004	scaling and root planing and chlorhexidine mouthrinses in the treatment of chronic periodontitis: a randomized, placebo-controlled clinical trial bana test; chlorhexidine; dental plaque; periodontal disease; scaling and root planing objective: evaluation of the clinical and microbiological effects of scaling and root planing (srp) alone or in combination with 0.12% chlorhexidine (chx) rinsing. methods: a blind, placebo-controlled, parallel-design, randomized clinical trial was conducted in 29 subjects with chronic periodontitis. Subjects were assigned to two therapeutic groups: control (srp+placebo) and test (srp+chx during and up to 42 days post-therapy). clinical and microbiological [n-benzoyl-dl-arginine-2-naphthylamide (bana test)] examinations were performed at baseline, 42 and 63 days post-therapy. results: initially, intermediate sites (4-6 mm) in the test group showed less plaque accumulation, gingival bleeding, bleeding on probing and a greater reduction in attachment level and probing depth (pd) at 63 days after treatment. the initially deep sites (>6 mm) in the chx group also showed a better reduction in plaque accumulation and in pd compared with the control group. both therapies led to a microbiological improvement; however, the test subjects showed a higher frequency of bana-negative sites after treatment, which was sustained over time ($p < 0.001$). at 63 days, the control group presented 25 bana-negative sites and 65 positive sites, and the test group 58 and 26, respectively. conclusion: the combination of chx rinses and srp leads to clinical benefits and to a better reduction in bana-positive species.	day (45.3, 1.0E-4); treatment (45.3, 1.0E-4); reduction (36.98, 1.0E-4); bana-negative site (30.16, 1.0E-4); scaling (30.16, 1.0E-4)	oral microbiome (0.03); dental plaque (0.02); oral

false	35	7	0.998	2003	a preliminary study of the effect of probiotic streptococcus salivarius k12 on oral malodour parameters halitosis; oral malodour; probiotics; streptococcus salivarius aims: to determine whether dosing with bacteriocin-producing streptococcus salivarius following an antimicrobial mouthwash effects a change in oral malodour parameters and in the composition of the oral microbiota of subjects with halitosis. materials and results: twenty-three subjects with halitosis undertook a 3-day regimen of chlorhexidine (chx) mouth rinsing, followed at intervals by the use of lozenges containing either s. salivarius k12 or placebo. assessment of the subjects' volatile sulphur compound (vsc) levels 1 week after treatment initiation showed that 85% of the k12-treated group and 30% of the placebo group had substantial (> 100 ppb) reductions. the bacterial composition of the saliva was monitored by culture and pcr-denaturing gradient gel electrophoresis (pcr-dgge). changes in the pcr-dgge profiles occurred in most subjects following k12 treatment. in vitro testing showed that s. salivarius k12 suppressed the growth of black-pigmented bacteria in saliva samples and also in various reference strains of bacteria implicated in halitosis. conclusions: administration of bacteriocin-producing s. salivarius after an oral antimicrobial mouthwash reduces oral vsc levels. significance and impact of the study: the outcome of this preliminary study indicates that the replacement of bacteria implicated in halitosis by colonization with competitive bacteria such as s. salivarius k12 may provide an effective strategy to reduce the severity of halitosis.	halitosis (59.74, 1.0E-4); salivarius k12 (29.79, 1.0E-4); change (25.98, 1.0E-4); salivarius (24.26, 1.0E-4); bacteria (23.07, 1.0E-4)	oral microbiome (0.03); dental plaque (0.02); oral
false	37	7	0.995	2007	antimicrobial activity of citrox® bioflavonoid preparations against oral microorganisms background citrox (r) is a formulation of soluble bioflavonoids obtained from citrus fruits. the non-toxic and antimicrobial properties of natural bioflavonoids are well documented, and consequently there has been interest in the therapeutic application of these substances. objective to determine the antimicrobial activity of two citrox (r) formulations (bc30 and mdc30) with different	formulation (77.62, 1.0E-4); bacterial species (30.9, 1.0E-4); citrus fruit (15.43, 1.0E-4); bioflavonoid preparation (15.43, 1.0E-4); broad-spectrum	oral microbiome (0.03); dental plaque (0.02); oral

					bioflavonoid combinations against a range of oral microorganisms. methods the antimicrobial activity of both formulations was tested against 14 bacterial species and six candida species. the two citrox (r) formulations (dilution range 0.007-8% v/v) were firstly evaluated by determining the in vitro minimal inhibitory concentration (mic) against planktonic microorganisms in a broth microdilution assay. secondly, the ability of the same serial dilutions to inhibit microbial growth was assessed in a modified microtitre biofilm assay. results both citrox (r) formulations exhibited antimicrobial activity. the bc30 formulation demonstrated greater activity than mdc30 and significantly inhibited growth of all bacterial species and most candidal species tested at a concentration of 1% (v/v) in both the broth and the biofilm assay. conclusion bioflavonoid preparations of citrox (r) have a broad-spectrum of antimicrobial activity against oral microorganisms, and as such have the potential to be used within therapeutic preparations for the control of the oral microflora.	(15.43, 1.0E-4)	
false	40	7	0.998	2009	biofilm formation on composite resins for dental restorations: an in situ study on the effect of chlorhexidine mouthrinses chlorhexidine; composites; biofilm; dental plaque; caries; silorane purpose: biofilm formation on the surface of dental restorative materials by oral bacteria is considered an important step in the development of secondary caries. the aim of this study was to evaluate the in situ effect of a chlorhexidine (chx)-containing mouthrinse on the biofilm formation occurring on the surface of human enamel and of two resin-based commercially available materials: a silorane-based material (filtek silorane (r)) and a methactylate-based material (filtek supreme xt (r)). methods: 53 disks were obtained for each of the two composites and 37 disks for enamel. the surface was characterized by determining the surface roughness and the surface free energy of 5 samples for each of the three materials tested, then the remaining samples were mounted on splints worn by 16 volunteers. the participants were randomly divided into two groups: an experimental group that used 0.12% chx-based mouthrinse	surface (73.76, 1.0E-4); placebo mouthrinse (34.6, 1.0E-4); biofilm formation (31.96, 1.0E-4); disk (29.4, 1.0E-4); group (29.4, 1.0E-4)	oral microbiome (0.03); dental plaque (0.02); oral

					and a control group that used a placebo mouthrinse. biofilm formation on the different surfaces after a 24 h period was assessed using mtt assay. results: the two composites in the group treated with the placebo mouthrinse showed a similar biofilm formation, which was significantly higher than that occurring on enamel surfaces. the chx-based mouthrinse significantly reduced biofilm formation on the surfaces of the two resin-based materials when compared with the placebo mouthrinse. the reduction was particularly relevant on the filtek silorane surfaces. conclusions: the new silorane-based material seems to interact with chx in a promising way from the point of view of biofilm formation<br>control.		
false	30	6	0.998	2007	effect of six different peri-implantitis disinfection methods on in vivo human oral biofilm antimicrobial agent; implant surface; oral bacteria; peri-implantitis objective the aim of this human in vivo pilot study was to evaluate the efficacy of six antimicrobial agents on the surface decontamination of an oral biofilm attached to titanium implants. design for in vivo biofilm formation, we fixed titanium specimens to individual removable acrylic upper jaw splints (14 specimens in every splint), which were worn by four volunteers overnight for 12 h. the specimens were then treated with different antimicrobial agents for 1 min (sodium hypochlorite, hydrogen peroxide 3%, chlorhexidingluconate 0.2%, plax, listerine, citric acid 40%). afterwards, we quantified the total bacterial load and the viability of adhering bacteria by live or dead cell labelling in combination with fluorescence microscopy. results the total bacterial load on the titanium surfaces was significantly higher after incubation in the control solution phosphate-buffered saline (pbs) than after disinfection in sodium hypochlorite, hydrogen peroxide, chlorhexidine, plax, listerine, and citric acid. furthermore, a significantly lower ratio between dead and total adhering bacteria (bactericidal effect) was found after incubation in control pbs, plax mouth rinse, and citric acid than after incubation in sodium hypochlorite, hydrogen peroxide, chlorhexidine, and	incubation (45.3, 1.0E-4); citric acid (45.3, 1.0E-4); total bacterial load (30.16, 1.0E-4); listerine (30.16, 1.0E-4);<br>titanium surface (30.16, 1.0E-4)	oral microbiome (0.03); dental plaque (0.02); oral

					listerine. conclusions all tested antiseptics seem to be able to reduce the total amount of microorganisms accumulating on titanium surfaces. furthermore, sodium hypochlorite, hydrogen peroxide, chlorhexidine, and listerine showed a significant bactericidal effect against adhering bacteria. to cite this article:gosau m, hahnel s, schwarz f, gerlach t, reichert te, burgers r. effect of six different peri-implantitis disinfection methods on in vivo human oral biofilm.clin. oral impl. res. 21, 2010; 866-872.doi: 10.1111/j.1600-0501.2009.01908.x.		
false	44	6	0.998	2009	antibacterial effects of blackberry extract target periodontopathogens antibacterial; antimicrobial; blackberry; periodontitis;periodontopathogens; fusobacterium background and objective: antimicrobial agents provide valuable adjunctive therapy for the prevention and the control of oral diseases. limitations in their prolonged use have stimulated the search for new, naturally occurring agents with more specific activity and fewer adverse effects. here we sought to determine the antibacterial properties of blackberry extract (bbe) in vitro against oral bacterial commensals and periodontopathogens. material and methods: the effects of whole and fractionated bbe on the metabolism of 10 different oral bacteria were evaluated using the colorimetric water-soluble tetrazolium-1 assay. the bactericidal effects of whole bbe against fusobacterium nucleatum were determined by quantitating the numbers of colony-forming units (cfus). cytotoxicity was determined in oral epithelial (okf6) cells. results: bbe at 3501400 mu g/ml reduced the metabolic activity of porphyromonas gingivalis, f. nucleatum and streptococcus mutans. the reduced metabolic activity observed for f. nucleatum corresponded to a reduction in the numbers of cfus following exposure to bbe for as little as 1 h, indicative of its bactericidal properties. an anthocyanin-enriched fraction of bbe reduced the metabolic activity of f. nucleatum, but not of p. gingivalis or s. mutans, suggesting the contribution of species-specific agents in the whole bbe. oral epithelial cell viability was not reduced following exposure to whole bbe (2.241400 mu g/ml) for = 6	bbe (59.07, 1.0E-4); nucleatum (44.26, 1.0E-4); whole bbe (44.26, 1.0E-4); prevention (29.46, 1.0E-4); blackberry (29.46, 1.0E-4)	oral microbiome (0.03); dental plaque (0.02); oral

					h. conclusion: bbe alters the metabolic activity of oral periodontopathogens while demonstrating a minimal effect on commensals. the specific antibacterial properties of bbe shown in this study, along with its previously demonstrated anti-inflammatory and antiviral properties, make this natural extract a promising target as an adjunct for prevention and/or complementary therapy of periodontal infections.		
false	46	6	0.998	2013	dental plaque bacteria with reduced susceptibility to chlorhexidine are multidrug resistant chlorhexidine; chryseobacterium culicis; chyseobacterium indologenes;antimicrobial resistance background: chlorhexidine (chx) is used in oral care products to help control dental plaque. in this study dental plaque bacteria were grown on media containing 2 mu g/ml chlorhexidine gluconate to screen for bacteria with reduced chx susceptibility. the isolates were characterized by 16s rrna gene sequencing and antibiotic resistance profiles were determined using the disc diffusion method. results: the isolates were variably resistant to multiple drugs including ampicillin, kanamycin, gentamicin and tetracycline. two species, chryseobacterium culicis and chryseobacterium indologenes were able to grow planktonically and form biofilms in the presence of 32 mu g/ml chx. in the chx and multidrug resistant c. indologenes we demonstrated a 19-fold up-regulation of expression of the hlyd-like periplasmic adaptor protein of a tripartite efflux pump upon exposure to 16 mu g/ml chx suggesting that multidrug resistance may be mediated by this system. exposure of biofilms of these resistant species to undiluted commercial chx mouthwash for intervals from 5 to 60 s indicated that the mouthwash was unlikely to eliminate them from dental plaque in vivo. conclusions: the study highlights the requirement for increased vigilance of the presence of multidrug resistant bacteria in dental plaque and raises a potential risk of long-term use of oral care products containing antimicrobial agents for the control of dental plaque.	exposure (30.23, 1.0E-4); biofilm (23.51, 1.0E-4); presence (22.6, 1.0E-4); ml chx (22.6, 1.0E-4); oral care product (21.24, 1.0E-4)	oral microbiome (0.03); dental plaque (0.02); oral
