# Supplementary material for: Research Trends in Antimicrobial Oral Hygiene Products, the Oral Microbiome, and Dental Biofilm: A Bibliometric Analysis (2006–2025)
Source: Antibiotics (Basel). 2026 Aug 29;15(9):839. doi: 10.3390/antibiotics15090839 (PMC13603143; doi:10.3390/antibiotics15090839)
Supplement: Supplementary file 1 [file antibiotics-15-00839-s001.zip › Supplementary Table S2.pdf]

Supplementary Table S2. Methodological configuration and algorithmic parameters of the bibliometric analysis platforms.

Software	Analysis Type	Node Type / Metadata Field	Selection Criteria & Thresholds	Normalization / Network Optimization	Clustering & Labeling Algorithms
**VOSviewer**	Social Collaboration Networks	Authors, Countries, Institutions	Minimum productivity: ≥ 3 documents per node	Association Strength	Default VOS clustering algorithm
**VOSviewer**	Conceptual Structure	Author Keywords	Minimum frequency: ≥ 6 occurrences (Yielded 89/1,773 terms)	Association Strength	VOS clustering (Partitioned into 7 distinct clusters)
**CiteSpace**	Structural & Intellectual Shifts	References (Co-citation)	Top 50 records per individual slice (N = 50)	Pathfinder pruning algorithm	Log-Likelihood Ratio (LLR) from citing titles
**CiteSpace**	Active Research Fronts	References (Citation Bursts)	Kleinberg's burst-detection algorithm (Top 25 bursts up to 2025)	–	–
**Bibliometrix**	Source Structures & Ranking	Scientific Journals	Bradford's Law of Scattering (Core Zone 1)	–	–
**Bibliometrix**	Research Trajectories	Author Keywords	Word frequency distribution across publication years	Trend Topic plotting	–
**Bibliometrix**	Longitudinal Evolution	Keywords (Co-word nexus)	4 document-balanced periods: 2006–14, 2015–19, 2020–22, 2023–25	Alluvial / Sankey diagram mapping	Callon's Centrality (X-axis) and Density (Y-axis)
**Bibliometrix**	Strategic Maps	Keywords (Co-word nexus)	Evaluated within the 4 chronological sub-periods	–	Strategic quadrant clustering (Motor, Niche, Basic, Emerging/Declining)
